# Explainable deep learning for predicting temperature breaks and identifying their causal drivers in fresh produce cold chains

**DOI:** 10.3389/frai.2026.1893108

**Published:** 2026-10-27

**Authors:** Jeremiah Taguta, Jean Frederic Isingizwe Nturambirwe, Clement Nthambazale Nyirenda

**Affiliations:** 1 Department of Computer Science, University of the Western Cape, Cape Town, South Africa; 2 eResearch Office, University of the Western Cape, Cape Town, South Africa

**Keywords:** causal discovery, food wastage, fresh fruits and vegetables, machine learning, supply chain, temperature break prediction, temperature prediction

## Abstract

**Introduction:**

This study proposes an integrated framework combining deep learning, causal discovery, explainability, and domain thresholds for proactive monitoring in temperature-sensitive fresh fruits and vegetables cold chains, a domain that remains largely unexplored. The framework predicts temperature breaks (a critical physical hazard that compromises food safety and accelerates postharvest losses), prospectively identifies their causal drivers, and prioritizes countermeasures.

**Methods:**

Using two IoT-gathered datasets from an Apple cold room in South Africa, eight machine learning models and three baselines were compared for temperature prediction, including a proposed LSTM-GRU hybrid (LSGR) model. Causal relationships between measured parameters and predicted temperature were identified using Latent Peter & Clark Momentary Conditional Independence (LPCMCI; compared with PCMCI). At the same time, SHapley Additive exPlanations quantified feature importance, and domain-based thresholds complemented them for countermeasure prioritization after temperature-break prediction (TBP).

**Results:**

On the extended dataset, deep neural networks' (DNN) *R*^2^ and normalized mean squared error improved by up to 30% and to 61%, respectively, over the smaller dataset, demonstrating strong generalizability to substantially different cold room operating conditions. The LSGR demonstrated the best overall performance across both temperature and TBP tasks among the recurrent models assessed, raising the fewest false alarms per day. All DNNs flagged breaks in advance; no baseline did. LPCMCI recovered most direct causal links after break prediction, especially for lagged temperature, identifying it as the dominant factor associated with predicted breaks. Notably, post-TBP, causality, importance, and thresholds strongly agreed on the same top two critical parameters or countermeasures (temperature and humidity), providing consistent and actionable insights for decision-making.

**Discussion:**

These findings demonstrate the viability of combining causal discovery, explainability and thresholds for cold chain management, with implications for reducing food wastage and enhancing quality, safety, and sustainability from farm to fork in temperature-sensitive FFVs supply chains. LPCMCI recovered a few links overall, indicating the influence of unmeasured drivers and warranting further investigation, including additional features, their interactions, and seasonal data, as well as a fuller characterization of causality-regime dependence.

## 1 Introduction

Global food systems lose approximately 1.3 billion tons of food annually (a third), with fresh fruits and vegetables (FFVs) accounting for 38% of this waste, despite the capacity to feed 1.2 billion people (WWF South Africa, 2017; Goedhals-Gerber et al., 2024; Bai et al., 2023). Post-harvest losses during cold storage and distribution are a key contributor, responsible for 20%–44% of FFV losses in South Africa, where apples hold significant economic and export value (Goedhals-Gerber et al., 2024; Fourie et al., 2023; WWF South Africa, 2017; Fruit South Africa, 2024). This study, therefore, targets cold storage as a critical intervention point for reducing food waste (Bai et al., 2023; WWF South Africa, 2017). FFVs are nutrient-rich but highly perishable and temperature-sensitive, requiring precise cold chain control (Wang et al., 2024; Badia-Melis et al., 2018; FAO, 2019; Findlay and Combrink, 2013), as suboptimal temperatures and prevalent temperature breaks in developing countries, even brief ones, compromise quality, safety, and shelf life, risking consignment rejection, food-borne illness, or death (Goedhals-Gerber et al., 2024; Badia-Melis et al., 2018; Taguta et al., 2026b; Ndraha et al., 2018). IoT monitoring lacks predictive capability (Badia-Melis et al., 2018; Taguta et al., 2026b), which ML addresses by forecasting temperature trends from sensor data to enable proactive interventions (Badia-Melis et al., 2018; Taguta et al., 2026b), enhancing decision-making, supporting sustainable development goals on food security, reduced post-harvest loss, resource efficiency, and climate action (FAO, 2019; Taguta et al., 2026b).

Many studies have been conducted to predict temperature using ML, but they have not focused on the FFV cold chain (Ghoroghi et al., 2023; Hoang et al., 2021; Konovalenko et al., 2021; Machacuay et al., 2024; Mangini et al., 2020). Several studies have been conducted to predict upcoming temperatures in the cold chain of FFVs (Guo et al., 2024; Loisel et al., 2022; Badia-Melis et al., 2016; Jiang et al., 2023; Zou et al., 2023; Ayanoglu and Uysal, 2023; Haider et al., 2024). However, these studies only predicted temperature values and not temperature breaks, leaving room for stakeholders to classify whether the value is a break or not, a process which may result in incorrect judgement, causing temperature abuse on FFVs, though temperature break prediction (TBP) was encouraged in (Taguta et al. 2025, 2026a); Loisel et al. (2021); Taguta et al. (2026b). Only one study predicted the temperature and time to temperature breaks in cold rooms (Guo et al., 2024). However, the study did not predict cold breaks, as this is a classification task requiring classification metrics such as Precision and Recall, which were not used (Taguta et al., 2026b). It is also unclear how the "correct" and "incorrect" classes in Haider et al. (2024) were generated after predicting temperature values; thus, the classes cannot be concluded to correspond to temperature breaks. Moreover, the study did not compare the models in terms of regression and classification performance to determine whether better regression performance leads to better classification, as encouraged in Taguta et al. (2026b). Some studies detected temperature breaks (Meng et al., 2024; Xie et al., 2025), as well as their pre-defined causes (door openings and power failures) (Swain and Jenamani, 2022, 2023a,b). However, they did not predict temperature breaks or their causes, although both are encouraged in (Taguta et al., 2026a,b; Loisel et al., 2021).

Moreover, none of these studies (Guo et al., 2024; Loisel et al., 2022; Badia-Melis et al., 2016; Jiang et al., 2023; Zou et al., 2023; Ayanoglu and Uysal, 2023; Haider et al., 2024; Taguta et al., 2026a) used SHapley Additive exPlanations (SHAP) or its alternatives, which helps to make models' results transparent (Ahmed et al., 2025) by quantifying correlation between features and the predicted outcome. The temperature break causes prediction was also not done in previous studies (Guo et al., 2024; Loisel et al., 2022; Badia-Melis et al., 2016; Jiang et al., 2023; Zou et al., 2023; Ayanoglu and Uysal, 2023; Haider et al., 2024; Taguta et al., 2026a) as identified in Taguta et al. (2026b). ML models may predict from a feature, but the feature-to-target relationship might not be causal (Runge et al., 2019a, 2023), yet approaches like Peter & Clark Momentary Conditional Independence (PCMCI) and Latent Peter & Clark Momentary Conditional Independence (LPCMCI) deduct time-series causal relationships from observed data (Gerhardus and Runge, 2020; Runge et al., 2019b). Thus, existing studies lack a framework that prospectively identifies the causal drivers of predicted temperature breaks, which is important as it informs stakeholders on the future status of the cold room, with detailed information on the causes of any predicted break as well as countermeasures and their order of importance, so that they don't guess the remedy, which is error-prone, promoting accurate proactive control. Such a framework is encouraged in Haider et al. (2024) and Taguta et al. (2026a,b).

Most ML studies on FFV cold-chain temperature monitoring used basic or standalone sensors (Guo et al., 2024; Loisel et al., 2022; Badia-Melis et al., 2016; Jiang et al., 2023; Zou et al., 2023), with limited adoption of Wireless Sensor Networks (WSNs). IoT-collected data for temperature prediction remains rare (Jiang et al., 2023; Taguta et al., 2026a), and no studies have applied it to TBP. This is despite IoT providing autonomous, real-time data collection with reduced measurement errors and manual labor, capabilities that directly enhance the quality of the ML model training (Taguta et al., 2026b; Badia-Melis et al., 2016; Guo et al., 2024; Jiang et al., 2023). Most studies (Jiang et al., 2023; Ayanoglu and Uysal, 2023; Haider et al., 2024; Guo et al., 2024) focused on other fruits, thus cannot be generalized for apples, as each fruit has different characteristics and requires a customized cold chain (Ahmadzadeh et al., 2023; Badia-Melis et al., 2018). Only a few studies (Loisel et al., 2022; Badia-Melis et al., 2016; Guo et al., 2024; Taguta et al., 2026a) focused on the cold room which has different parameter settings compared to refrigerated transport (RT) (Department of Agriculture, Land Reform and Rural Development, 2001; Findlay and Combrink, 2013); of these, only three (Loisel et al., 2022; Badia-Melis et al., 2016; Taguta et al., 2026a) focused on apples, but only one of them (Taguta et al., 2026a) focussed on the South African supply chain, whose environment is unique, including systematic load-shedding which disrupt cold chains (Wright et al., 2024). This is despite South Africa being a major apple producer and exporter (Fruit South Africa, 2024). Thus, more cold-room-focused studies on apples are required, particularly in the South African context.

Moreover, the ML models in the reviewed literature (Guo et al., 2024; Loisel et al., 2022; Badia-Melis et al., 2016; Jiang et al., 2023; Zou et al., 2023; Ayanoglu and Uysal, 2023; Haider et al., 2024) had limited input parameters and no study incorporated additional environmental and physical parameters, namely pressure, humidity, CO_2_ concentration, ambient light, and vibration induced by acceleration, all of which are known to influence temperature dynamics in a cold chain. While Taguta et al. (2026a) included most of these parameters, the study focused on simulated deep neural network (DNN) model deployments in the fog and cloud, not on TBP and prospective causal driver identification, and had a different preprocessing pipeline, including simulated packet loss, which could affect the evaluation of the parameters' effect on ML performance. From the reviewed studies, no prior apples cold room study has examined the sensitivity of ML models to feature combinations to identify the minimum features required for reliable, cost-effective predictions, nor addressed the impact of data-splitting strategies important for handling overfitting (Zou et al., 2023). This, therefore, needs to be considered on different ML algorithms and a dataset of a different fruit. Additionally, dataset sensitivity analysis was not implemented in any of the reviewed studies to evaluate generalizability. Sensitivity analysis of ML models to the number of past readings, which is used to predict future value and affect accuracy (Machacuay et al., 2024; Xie et al., 2025), was not implemented in any of the previous studies focusing on TP and TBP in the FFVs cold chain (Guo et al., 2024; Loisel et al., 2022; Badia-Melis et al., 2016; Jiang et al., 2023; Zou et al., 2023; Ayanoglu and Uysal, 2023; Haider et al., 2024; Taguta et al., 2026a).

Gated recurrent units (GRUs) and long short-term memory (LSTM) were not used in Loisel et al. (2022); only multi-layer perceptron (MLP) was used in Badia-Melis et al. (2016), Zou et al. (2023) used no GRUs and deep LSTMs, Ayanoglu and Uysal (2023) used only convolutional neural network (CNN), Haider et al. (2024) only used traditional ML models, (Jiang et al. 2023) used no GRUs and deep LSTM, (Guo et al. 2024) used only a deep LSTM. Some studies did not compare traditional ML models (Badia-Melis et al., 2016; Zou et al., 2023; Ayanoglu and Uysal, 2023; Jiang et al., 2023; Guo et al., 2024) with GRUs and LSTMs. Among the reviewed studies, none used an extreme gradient boosting (XGB), which is recognized for speed, scalability, and precision (Nturambirwe and Opara, 2020); bidirectional GRU (bGRU) or bidirectional LSTM (bLSTM), preferred for high performance, as well as an LSTM-GRU (LSGR) hybrid, combining LSTM's long-term memory with GRU's computational efficiency (Ghoroghi et al., 2023; Hoang et al., 2021). Therefore, a study comparing most of these models on the same dataset is necessary.

The preliminary version of this study was presented and published at a conference (Taguta et al., 2025). The study focused on the South African apple cold room, comparing only the temperature predictive performance of recurrent neural networks (GRU) with non-recurrent networks, and back-propagation-based models (GRU and MLP) with feature-mapping models (extreme learning machines (ELM) and XGB). This study extends prior studies, also focusing on the South African Apple cold chain, by comparing the TP performance of the models used in Taguta et al. (2025) with additional DNN models (LSGR, stacked LSTM (sLSTM), bGRU, and bLSTM) on a smaller, extended dataset. TP and TBP, incorporating additional environmental parameters, are conducted using IoT-gathered data, and also comparing models' TP and TBP performances. ML is integrated with PCMCI and LPCMCI to prospectively identify the causal drivers of future temperature. The measured parameters' causality and SHAP importance to predicted breaks and the magnitude of deviation are used to provide prioritized countermeasures for predicted breaks. This enables proactive FFVs temperature abuse prevention, reducing wastage and enhancing food safety and security, making the following contributions:

- Design and evaluation of deep learning models for predicting internal cold room temperature and temperature breaks under a dual regression-classification framework.
- Generalization of proposed and existing temperature prediction ML models is assessed by extending evaluation to a richer extended dataset (EDS), with sensitivity analysis across feature combinations, temporal windows, and data-splitting strategies.
- A comparison of features' causality (LPCMCI vs PCMCI) and importance (SHAP) across predicted temperatures and temperature breaks, with the latter incorporating a safety-based regulatory threshold approach for feature prioritization.
- A multi-layer explainability framework linking causal drivers (LPCMCI), deviation triggers, and SHAP contributions to help stakeholders understand, prioritize, and act on predicted temperature breaks.

the rest of the paper is organized as follows: section 2 describes ML models used in the current study, section 3 presents the proposed framework, Section 4 describes the experimentation, Section 5 presents the results and discussions and Section 6 covers the conclusions.

## 2 Overview of ML models used

This study employs multi-layer perceptron (MLP), extreme learning machines (ELM), extreme gradient boosting (XGB), and traditional gated recurrent unit (tGRU) used in Taguta et al. (2025) and adds stacked long short-term memory (sLSTM), bidirectional LSTM (bLSTM), bidirectional GRU (bGRU), and LSTM-GRU (LSGR) hybrid models, as discussed below.

### 2.1 Extreme gradient boosting

It is an ensemble learning method based on gradient boosting (Equations 1–4), in which a strong predictive model is formed by successively adding weak learners, often decision trees, where each tree corrects the residual errors of the previous ensemble (Chen and Guestrin, 2016). An XGB is preferred in this study for its reliability, speed, scalability, and accuracy, employing gradient boosting and decision tree ensembles (Hassan et al., 2023; Chen and Guestrin, 2016). Given a dataset of *N* samples with *n* features, the predicted output at boosting iteration *t* is given by:


$$\begin{array}{l} {ŷ}_{k}^{\left(t\right)}={ŷ}_{k}^{\left(t-1\right)}+f_{t}\left({\text{x}}_{k}\right), \end{array}$$ (1)

where ${ŷ}_{k}^{\left(t-1\right)}$ is the prediction from the previous iteration and *f*_*t*_(**x**_*k*_) is the newly added tree. The objective function minimized at each iteration consists of a loss term and a regularization, as defined by:


$$\begin{array}{l} L^{\left(t\right)}=\overset{N}{\underset{k=1}{\sum }}l\left(y_{k},{ŷ}_{k}^{\left(t-1\right)}+f_{t}\left({\text{x}}_{k}\right)\right)+\Omega \left(f_{t}\right), \end{array}$$ (2)

where *l*(·) is a differentiable loss function, and Ω(*f*_*t*_) is the regularization term defined by:


$$\begin{array}{l} \Omega \left(f_{t}\right)=\gamma T+\frac{1}{2}\lambda ||\text{w}|{|}^{2}, \end{array}$$ (3)

where *T* is the number of leaves in the tree, **w** is the vector of leaf weights, γ controls the minimum gain required to split a node, and λ is the L2 regularization coefficient, which distinguishes XGBoost from standard gradient boosting and helps prevent overfitting. The optimal leaf weight for each node *j* is given by:


$$\begin{array}{l} w_{j}^{*}=-\frac{G_{j}}{H_{j}+\lambda }, \end{array}$$ (4)

where $G_{j}=\underset{k\in j}{\sum }\partial_{ŷ}l\left(y_{k},{ŷ}_{k}\right)$ and $H_{j}=\underset{k\in j}{\sum }\partial_{ŷ}^{2}l\left(y_{k},{ŷ}_{k}\right)$ are the first- and second-order gradient statistics over all samples in leaf *j*.

### 2.2 Multi-layer perceptron

The MLP is a feed-forward neural network (FFNN) comprising an input layer, one or more hidden layers, and an output layer (Rumelhart et al., 1986). Each neuron receives a weighted sum of outputs from the previous layer, passed through a non-linear activation function. For a single-hidden-layer architecture, the output of the hidden layer is defined by:


$$\begin{array}{l} h_{j}=f\left(\overset{n}{\underset{i=1}{\sum }}w_{ij}x_{i}+b_{j}\right), \end{array}$$ (5)

where *x*_*i*_ is the *i*-th input feature, *w*_*ij*_ is the weight connecting input *i* to hidden neuron *j*, *b*_*j*_ is the bias, and *f*(·) is the activation function. The predicted output (temperature in this case) is defined by:


$$\begin{array}{l} ŷ=g\left(\overset{m}{\underset{j=1}{\sum }}v_{j}h_{j}+b_{o}\right), \end{array}$$ (6)

where *v*_*j*_ are the output layer weights, *b*_*o*_ is the output bias, and *g*(·) is the output activation function, taken as linear for regression tasks. The network, governed by Equations 5–6, is trained by minimizing the loss function using back-propagation and gradient descent, making it preferred for efficiently handling complex problems and approximating continuous functions (Hassan et al., 2023).

### 2.3 Extreme learning machines

ELM, governed by Equations 7–9, is a form of FFNN with a three-layer architecture (input, single-hidden, and output) preferred in this study for its fast learning and good generalization capabilities due to randomly assigning the input weights and biases and analytically computing only the output weights, eliminating the need for iterative tuning (Huang et al., 2006). For Ñ hidden neurons and a training set of *N* samples, the hidden layer output, *h*_*i*_(**x**), for input **x**, is defined by:


$$\begin{array}{l} h_{i}\left(\text{x}\right)=f\left({\text{w}}_{i}\cdot \text{x}+b_{i}\right),\;i=1,2,\ldots ,Ñ, \end{array}$$ (7)

where **w**_*i*_ and *b*_*i*_ are the randomly initialized input weights and bias of the *i*-th hidden neuron. The predicted output, ŷ, temperature in this case, is defined by:


$$\begin{array}{l} ŷ=\text{H}\beta , \end{array}$$ (8)

where **H** is the hidden layer output matrix and ***β*** is the output weight vector. Since the input weights are fixed, the output weights are obtained by:


$$\begin{array}{l} \beta ={\text{H}}^{†}\text{Y}, \end{array}$$ (9)

where **H**^†^ is the Moore-Penrose pseudo-inverse of **H**.

### 2.4 Gated recurrent units

GRUs are a type of recurrent neural network that can handle sequential data, such as time-series forecasting, because they mitigate vanishing gradient problems and capture long-range dependencies. GRUs are useful for this study because of their well-known simplicity and speed of convergence, as well as their efficacy and outstanding performance in long-term pattern prediction, even for small datasets (Taguta et al., 2025; Ghoroghi et al., 2023). The GRU simplifies the LSTM by replacing the three-gate mechanism with two gates, a reset gate and an update gate, while eliminating the separate cell state (Cho et al., 2014), reducing model complexity while retaining the ability to learn long-range dependencies. A single-layer GRU (tGRU) processes sequences in a single temporal direction. At each time step *t*, the gates are computed as:


$$\begin{array}{l} r_{t}=\sigma \left(W_{r}\left[h_{t-1},x_{t}\right]+b_{r}\right) \end{array}$$ (10)

$$\begin{array}{l} z_{t}=\sigma \left(W_{z}\left[h_{t-1},x_{t}\right]+b_{z}\right), \end{array}$$ (11)

where *r*_*t*_ is the reset gate and *z*_*t*_ is the update gate. The candidate's hidden state is computed as:


$$\begin{array}{l} {\tilde{h}}_{t}=\tanh\left(W_{h}\left[r_{t}⊙h_{t-1},x_{t}\right]+b_{h}\right) \end{array}$$ (12)

where *r*_*t*_ controls how much of the previous hidden state is retained. The final hidden state is updated as:


$$\begin{array}{l} h_{t}=\left(1-z_{t}\right)⊙h_{t-1}+z_{t}⊙{\tilde{h}}_{t}, \end{array}$$ (13)

where *z*_*t*_ balances the contribution of the previous hidden state and the new candidate state. The temperature forecast ŷ is obtained by passing *h*_*t*_ through a fully connected output layer.

The bidirectional GRU (bGRU) follows Equations 10–13, processing the input sequence in both forward and backward directions within the given window, capturing both early and late temporal patterns in the historical observations, which makes them applicable in this study.

### 2.5 Long short-term memory

LSTMs are recurrent neural networks that feature a three-gate mechanism (forget, input, and output) explicitly designed to handle sequential data while mitigating the vanishing-gradient problem. Their capacity to model complex, non-linear temporal dependencies makes them well-suited for this forecasting task (Hoang et al., 2021; Hochreiter and Schmidhuber, 1997). The LSTM architecture is implemented following Hochreiter and Schmidhuber (1997) and Gers et al. (2000), with input, forget, and output gates regulating information flow through the cell state, *C*_*t*_. At each time step *t*, the gates are computed as:


$$\begin{array}{l} f_{t}=\sigma \left(W_{f}\left[h_{t-1},x_{t}\right]+b_{f}\right) \end{array}$$ (14)

$$\begin{array}{l} i_{t}=\sigma \left(W_{i}\left[h_{t-1},x_{t}\right]+b_{i}\right) \end{array}$$ (15)

$$\begin{array}{l} o_{t}=\sigma \left(W_{o}\left[h_{t-1},x_{t}\right]+b_{o}\right), \end{array}$$ (16)

where *f*_*t*_, *i*_*t*_, and *o*_*t*_ are the forget, input, and output gates, respectively; σ(·) is the gating activation function (typically sigmoid), *h*_*t*−1_ is the previous hidden state, and *W* and *b* denote the weight matrices and bias vectors. The cell state, *C*_*t*_, is updated as:


$$\begin{array}{l} C_{t}=f_{t}⊙C_{t-1}+i_{t}⊙\phi \left(W_{C}\left[h_{t-1},x_{t}\right]+b_{C}\right), \end{array}$$ (17)

where ⊙ denotes element-wise multiplication and ϕ is the cell activation function. The hidden state output, *h*_*t*_, is then defined as:


$$\begin{array}{l} h_{t}=o_{t}⊙\phi \left(C_{t}\right), \end{array}$$ (18)

where ϕ is the cell and hidden state activation function. The temperature forecast, ŷ, is obtained by passing *h*_*t*_ through a fully connected output layer.

Furthermore, a stacked LSTM (sLSTM) aggregates multiple recurrent layers to extract deep hierarchical features from the temporal data. In this architecture, the hidden state $h_{t}^{\left(l\right)}$ of the *l*-th layer serves as the input to the (*l*+1)-th layer. Each layer in the stack follows the standard LSTM formulation described in Equations 14–18, with the final prediction, ŷ, derived from the hidden state of the uppermost layer.

Bidirectional LSTM (bLSTM) follows Equations 14–18, processing the data in both forward and reverse directions strictly within the bounds of the input window (Hoang et al., 2021). This captures both early and late temporal patterns in the historical observations, which makes them applicable in this study.

An LSGR hybrid is well-suited for this study, as it combines LSTM and GRU architectures to balance long-term memory with computational efficiency for unstable cold-chain regimes. This approach has demonstrated robust predictive performance and resilience to packet loss in simulated fog-based cold chain deployments (Taguta et al., 2026a).

## 3 The proposed framework

This section outlines the framework proposed in this study, which includes the ML-based predictive engine; the explainable AI component, comprising causal discovery for prospective identification of predicted temperature break causal drivers, feature importance based on SHAP, and dynamic triggers based on Apple's cold-room safety regulatory thresholds; and counter-measure prioritization based on the three approaches.

### 3.1 Predictive modeling engine

The predictive engine includes temperature prediction and a transition to temperature break prediction. These components are described as follows:

#### 3.1.1 Temperature prediction: baseline and proposed models

Four optimized predictive configurations were developed and benchmarked: sLSTM, bLSTM, bGRU, and a reconfigured LSGR (Taguta et al., 2026a), described as follows: The sLSTM was configured with multiple stacked LSTM layers to enable hierarchical learning of temporal patterns in the temperature sequence. The bLSTM and bGRU were configured to process temperature sequences in both temporal directions within the input window, thereby capturing asymmetric temporal patterns in cold chain monitoring data. The LSGR hybrid consists of a GRU layer followed by a stacked LSTM layer, an arrangement determined empirically through experimentation. MLP, ELM, XGB, and tGRU proposed in Taguta et al. (2025) served as comparison baselines. The search space and final architecture details of the ML models are provided in Supplementary Tables S1 and S2, respectively.

#### 3.1.2 From temperature to temperature break prediction conversion

To predict temperature, and thereby predict temperature breaks, baseline tGRU and DNN models such as LSGR, sLSTM, bGRU, and bLSTM were used for their time-series analysis capabilities. The model's predicted and actual test data temperatures, *T*, were both converted to True (temperature breaks) when outside the –0.5 °C to 1°C range or False (No temperature breaks) if they fell within the range, inclusively. The temperature classes of *T* are defined by:


$$\begin{array}{l} \text{class}\left(T\right)=\{\begin{array}{cc} \text{False (No Break)} & \text{if}-0.5\leq T\leq 1.0\\ \text{True (Break)} & \text{if }T<-0.5\text{or}T>1.0. \end{array} \end{array}$$ (19)

Converting regression predictions to classification outputs is an established technique, as it was used in another study, which converted the results to "correct" or "incorrect," and then went on to apply classification metrics on the classified results; though the definition of these classes was not clearly defined as temperature breaks (Haider et al., 2024), as done in this study, based on domain knowledge.

### 3.2 Model explainability

To make the ML models' predictions transparent, SHapley Additive exPlanations (SHAP) importances and causal structures between the measured parameters and predicted temperatures were explored.

#### 3.2.1 SHAP-based feature importance

To convert models from black-box to transparent ones, SHAP was used (Ahmed et al., 2025) during the simulated real-time temperature prediction, displaying its values alongside other alerts. It is important to note that while SHAP values provide insight into a model's decision-making process, they reveal associational relationships and should not be interpreted as causal evidence (Ahmed et al., 2025; Docquier et al., 2024; Runge et al., 2023, 2019a). The application of SHAP is not only important but also key in understanding the key predictors of temperature, a driver of FFVs loss and waste, in the cold chain, which will inform future ML modeling on key features to utilize for accurate predictions.

#### 3.2.2 Time-series causal discovery: PCMCI and LPCMCI

Because feature-importance methods reflect association rather than causal influence, this study performs prospective causal attribution: causal drivers are identified from historical lagged dynamics and applied to predicted rather than realized breaks. The identified drivers are therefore candidate causal relationships supported by the observational data rather than experimentally verified mechanisms. Attribution is restricted to the four continuously monitored parameters (Temperature_Lagged, Humidity, Ambient Light, and CO_2_), which are the variables available for real-time intervention. Causal discovery is performed using Peter & Clark Momentary Conditional Independence (PCMCI) and Latent Peter & Clark Momentary Conditional Independence (LPCMCI). Both are suitable for time-series data and are based on the combination of the Peter & Clark algorithm (Spirtes et al., 2000) and momentary conditional independence (MCI) approaches. Both PCMCI and LPCMCI operate under standard time-series assumptions of stationarity, faithfulness, and the causal Markov condition. The key distinction is that PCMCI assumes causal sufficiency, that all relevant variables are observed, whereas LPCMCI extends PCMCI, relaxes this, handling latent (unobserved) confounders (Runge et al., 2019a, 2023, 2019b). Given that this study's IoT-based FFVs cold-chain monitoring included many unmeasured parameters, such as O_2_, external temperature, and humidity, among others, which may cause temperature changes or breaks, this relaxation motivates the comparison. Both are at the intersection of ML and statistics, learn the causal structure across past and present variables, and are particularly adept at identifying spurious relationships. For instance, CO_2_ and the predicted temperature may both change simultaneously. A naive method, such as PCMCI, might incorrectly infer that the most recent CO_2_ lag causes the temperature change. However, LPCMCI can detect if both variables are actually being driven by a common, unobserved factor, like door openings, and will therefore correctly identify the CO_2_ to temperature relationship as spurious and confounded rather than truly causal (Runge et al., 2019a, 2023). A feature's recent past observation is inferred to be causal to a predicted temperature if: (i) its *p*-value falls below the chosen significance threshold, indicating a statistically significant conditional dependency after controlling for the estimated causal history of all variables in the system; (ii) its absolute causal strength exceeds 0.1, confirming a meaningful effect size rather than mere statistical noise and (iii) LPCMCI or PCMCI assigns a directed link ( → ) from the feature to the target, confirming the direction of influence rather than simple association. Only when all three conditions are jointly satisfied is the feature considered a causal driver of the predicted temperature (Gerhardus and Runge, 2020; Runge et al., 2019b).

The LPCMCI and PCMCI require a conditional independence test, which depends on whether the variables are linearly or non-linearly correlated. The nature of the association was tested using Pearson correlation, with pairwise correlations of the measured parameters against the predicted temperature. Temperature_Lagged is significantly linearly correlated (0.918), while CO_2_ (0.669) and Humidity (0.559) are fairly linearly correlated, whereas ambient light (0.221) is not linearly correlated with the predicted temperature. Because most of the parameters under causal investigation are linearly correlated with the predicted temperature, ParCorr and RobustParCorr were used to determine which of the two approaches is best. Both ParCorr and RobustParCorr conditional independence tests were evaluated for their suitability with climate data. Given that climate data systematically exhibits non-Gaussian characteristics (Perron and Sura, 2013) and the importance of robust methods for reliable causal discovery in climate systems, the most appropriate method was selected based on performance evaluation. LPCMCI and PCMCI were compared using RobustParCorr to find the best approach (Gerhardus and Runge, 2020; Runge et al., 2023). These have not been used for discovering the causal relationship between cold chain parameters and predicted temperature or temperature breaks. LPCMCI has been used for river discharge causality analysis, while PCMCI has been used in climate studies for causal discovery. Both are recommended for climate causal analysis due to their complexity in climate studies (Gerhardus and Runge, 2020; Docquier et al., 2024), hence their usage in this study for causal discovery from FFVs cold-chain data. The PCMCI and LPCMCI usage in the FFVs cold chain adds to the body of knowledge on the main causal drivers of temperature breaks, based on observed data, in a domain where many products get easily spoiled and lost, negatively impacting food security, the economy and the environment. Causality comparison to SHAP's importance and threshold-based approach provides an opportunity to deeply analyze the FFVs cold chain to provide solutions for reduced wastage and increased security.

#### 3.2.3 Counter-measure prioritization

Feature scoring and alerting are presented as follows:

##### 3.2.3.1 Feature scoring using causality, importance, and deviation magnitude

A key contribution of this study is countermeasure (feature) prioritization, which integrates features' causality, importance to a predicted temperature break, as well as the degree of deviation. Though the approaches differ in what they measure, their integration provides an opportunity to get the best of all of them to inform stakeholders on the causes and priorities of countermeasures to maintain the quality and safety of FFVs while reducing their wastage.

The best TBP model was used to identify the causal drivers of a predicted break. It is a cause-prediction approach because the relationship between features and the predicted out-of-range temperature is discovered in advance, before the event occurs. This means that the cause predictions happened only if an out-of-range temperature was predicted (break), that is, below –0.5 °C or above 1 °C (Findlay and Combrink, 2013; Botes et al., 2019). The input vector, **x**_*t*_, is constructed by concatenating features from time steps *t*−1 and *t*, as defined by:


$$\begin{array}{l} \begin{array}{c} {\text{x}}_{t}=[x_{0,t-1},\ldots ,x_{N-1,t-1},\\ x_{0,t},\ldots ,x_{N-1,t}{]}^{⊤}\in {\mathbb{R}}^{2N}, \end{array} \end{array}$$ (20)

where *x*_*i, t*_ denotes the *i*-th feature at time step *t*, for *i* = 0, 1, …, *N*−1, and *N* is the total number of features. The feature vector (Equation 20) includes features of interest such as temperature (lag), relative humidity, CO_2_, ambient light, and others. These features are of interest because they have a huge effect on the upcoming internal temperature of FFVs and the cold room, indirectly affecting the shelf-life of FFVs (Findlay and Combrink, 2013). These were investigated because they are the measured parameters that can be controlled, whereas other features are interactions of the measured parameters. The acceptable cold storage conditions for most South African apple varieties are as follows: temperature between –0.5 °C and 1 °C, relative humidity between 90% and 95%, CO_2_ levels from 0% to 2.5%, and light levels at 0 lux (complete darkness) (Findlay and Combrink, 2013; Botes et al., 2019). The lower bound, *l*, and upper bound, *u*, are thus defined by:


$$\begin{array}{l} \text{l}={\left[-0.5,0.90,0,0\right]}^{⊤} \end{array}$$ (21)

and


$$\begin{array}{l} \text{u}={\left[1.0,0.95,25000,0\right]}^{⊤}, \end{array}$$ (22)

where bounds correspond to temperature (°C), relative humidity (expressed as a fraction), CO_2_ (ppm), and light (lux), respectively. The values ±0.0001 were used for the lower bounds for light and humidity and the upper bounds for light, instead of 0, to avoid division-by-0 errors.

Real-time temperature prediction was simulated by iterating through the feature test data to predict the temperature for the next hour, ${\hat{T}}_{t+1}$, defined by:


$$\begin{array}{l} {\hat{T}}_{t+1}=f_{T}\left({\text{x}}_{t-1},{\text{x}}_{t}\right), \end{array}$$ (23)

where *f*_*T*_ is the temperature prediction model.

To enable proactive identification of parameter deviations upon predicting deviated temperature (Equation 23), the environmental assessment framework, $R_{\text{env}}$(·), is defined by:


$$\begin{array}{l} R_{\text{env}}\left({\text{x}}_{t}\right)=\left\{p\in \left\{T,RH,CO_{2},L\right\}:x_{p}\notin \left[l_{p},u_{p}\right]\right\}, \end{array}$$ (24)

where **x**_*t*_ represents the current terminal sensor readings, which serve as initial conditions for the next iteration.

To identify which parameters would exceed acceptable ranges and determine the causes of predicted temperature breaks from the domain perspective, an assessment is done on the current feature vector's latest time step, **x**_*t*_, using $R_{\text{env}}\left({\text{x}}_{t}\right)$ (Equation 24). Each returned feature *x*_*i, t*_ represents a violation requiring intervention, with deviation magnitude, *d*, defined by:


$$d=\{\begin{array}{cc} \frac{\left|x_{i,t}^{l}-x_{i,t}\right|}{\left|x_{i,t}^{l}\right|}, & x_{i,t}<x_{i,t}^{l}\\ \frac{x_{i,t}-x_{i,t}^{u}}{x_{i,t}^{u}}, & x_{i,t}>x_{i,t}^{u}\\ 0, & \text{otherwise} \end{array}$$ (25)

where the superscripts *l* and *u* represent the feature's lower and upper bounds, defined in Equations 21, 22, respectively.

Additionally, each feature's intervention is assigned a priority score, *ps*, that emphasizes causal relationships, defined by:


$$\begin{array}{l} \text{ps}=\min\left(\text{b\_s}+C\cdot {\text{1}}_{\text{is\_causal}},100\right), \end{array}$$ (26)

where the constant *C* determines the bonus assigned to causal features. *C* is added when a feature is causal, placing every causal feature above every non-causal feature. Since the feature's base score, *b*_*s*, cannot exceed 1, any value *C*>1 yields an identical rank ordering. It implements a categorical tier separation rather than a weighted contribution. This study uses *C* = 15 so that the tier is visually apparent in reported scores. is_causal equals 1 if the feature is causal and 0 otherwise, according to the criteria in Section 3.2.2. The resulting priority score ranges from 0 to 100. b_*s*_ is defined as:


$$\begin{array}{l} \begin{array}{cc} \text{b\_s} & =0.7np+0.2s+0.1m, \end{array} \end{array}$$ (27)

where *s* is the causality strength from LPCMCI, and *m* is the SHAP importance value, all discussed in Section 3.2. The weights (0.7, 0.2, 0.1) are author-defined heuristics that encode the relative operational priority of the three score components. Normalized priority receives the highest weight because a deviated parameter is the most directly actionable; correcting it is what averts the predicted break. Causal strength is weighted second, as it identifies the potential causal drivers of that break. Model-derived importance receives the lowest weight because it reflects a feature's contribution to the model's prediction rather than its causal influence on the physical system. The weights therefore affect ordering within a tier, not tier assignment. They are not learned from data and may be reconfigured for a given facility. Validation against expert judgement or operational interventions was beyond the scope of this study; the prioritization scheme is therefore presented as a proof of concept. *np* is a normalized priority, defined by:


$$\begin{array}{l} \text{np}=\min\left(d,1\right), \end{array}$$ (28)

which is important since features have differing ranges and units.

##### 3.2.3.2 Alerting

After a deviated feature's priority score, *ps*, is computed based on Equation 26, it is assigned an alerting class, *alert*_*class* defined by:


$$\begin{array}{l} \text{alert\_class}=\{\begin{array}{cc} \text{CRITICAL} & \text{if}\text{np}\geq 0.7\text{ or }\text{ps}\geq \text{C},\\ \text{SEVERE} & \text{if}0.3<\text{np}<0.7,\\ \text{WARNING} & \text{if}0<\text{np}\leq 0.3, \end{array} \end{array}$$ (29)

where *np* and *ps* are defined in Equations 26, 28, respectively. The alert thresholds were selected by the authors to ensure that any causal feature, or a non-causal feature showing significant deviation, triggers an urgent call for action, while ensuring that slightly deviated and non-causal features may not cause panic among stakeholders. This approach ensures that environmental parameters that drive the temperature changes, which are important and exceed their optimal ranges, are flagged for immediate intervention. The stakeholder is informed on what to do, in which order and by how many units to reduce or increase a parameter setting (based on the deviation magnitude, *d*, defined in Equation **??**), thereby maintaining optimal storage conditions for apple preservation, which is a key contribution of this study.

## 4 Experimentation

This section outlines the approaches used for data collection, cleaning, feature engineering, optimization and explainability of ML models, sensitivity analysis, and performance metrics.

### 4.1 Data collection, cleaning, and feature engineering

Building on the study by Taguta et al. (2025), ambient temperature, humidity, pressure, CO_2_, light, and motion data were collected from an experimental cold room at an Agricultural Research Institute in the Western Cape, South Africa, containing 40 crates full of sundowner apples ( 4,000 fruits). LoRaWAN-based sensors, Synetica enLink Zone Plus and Netvox R718E, uploaded data to the cloud via the iFemtoCell gateway. Temperature, velocity, and acceleration were recorded by a Netvox R718E (basic error limit ±3 °C) placed inside a crate of apples, in the cold room; humidity, pressure, CO_2_, and ambient light by a Synetica enLink Zone Plus (temperature ±0.2 °C, humidity ±2%, CO_2_ ±(30 ppm + 3%)), initially positioned at the entrance and later moved inside and placed in a crate with apples. Its temperature was not used to avoid data leakage when it was located inside (Taguta et al., 2025).

As encouraged by Taguta et al. (2025), additional data from Apple's cold room were collected. This resulted in an extended temporal scope, as data collection spanned July 2024 to December 2024, compared to the Conference Paper (CP) dataset, which covered July to August 2024. This led to an increase in size as well, with the extended dataset (EDS) containing 3,968 rows after merging, a 310.8% increase over the CP's 966-row dataset (almost 4.1 times larger), improving statistical robustness for transient temperature modeling. This addresses data quantity limitations in prior studies (Jiang et al., 2023; Taguta et al., 2025). Additionally, the extension resulted in the data covering a broader temperature range, with the EDS reflecting a colder minimum (–0.2 °C vs CP's 0.5 °C) and a warmer maximum (30.2 °C vs. CP's 6.2 °C), capturing extreme cold-chain failure scenarios or temperature breaks, absent in the CP dataset. Temperature breaks were deliberately induced during data collection to cover the range of cold chain scenarios required for model training; extreme readings therefore reflect induced excursions rather than sensor anomalies or equipment failures. All break observations were upper-threshold excursions (*n* = 702); no lower-threshold breaches occurred, so separate lower-threshold performance could not be assessed. The pipeline (hourly alignment, leakage prevention, feature engineering, and mutual-information-based feature selection) was retained to ensure direct comparability (see Taguta et al., 2025 for preprocessing details). After aligning and merging columns of interest based on Supplementary Table S3, from the two sensors, 2.27% of the values were missing. These missing values were subsequently imputed using forward filling (Taguta et al., 2025). Feature selection was initially done based on MI scores (Huang et al., 2024) and domain knowledge-based trial and error, as in Taguta et al. (2025). Derived features were grouped by construction. Lagged features took the previous observation; rolling features were computed over trailing five-step windows [*t*−4, *t*]; rate-of-change features took first differences, with temperature differences from its lagged value (*temperature*_*t*−1_−*temperature*_*t*−2_); and interaction features were products of lagged terms at *t*−1. All are therefore backwards-looking, and no feature uses a statistic computed over the full series. Together with chronological splitting using TimeSeriesSplit, this ensures that no training observation depends on test-period data. To prevent data leakage, the MinMax scalers (Henderi et al., 2021) for features and the target were fitted on training data and applied unchanged to the test set. The final split produced by TimeSeriesSplit was used, covering 2024-07-19 to 2024-11-20 17:00:00+02:00 for training, with the final 20% reserved for validation, and 2024-11-20 18:00:00+02:00 to 2024-12-31 for testing. The test period contains 3 break events or episodes which lasted 56, 176, and 470 h (median 176 h, approximately 7 days), with a 71% break prevalence. Sequences were constructed after splitting, so no test window contains training observations.

### 4.2 ML Modeling

Machine learning models such as ELM, XGB, LSTMs, and GRUs were implemented using specialized libraries (hpelm, xgboost, and Keras). There were separate processes for standalone temperature prediction and for temperature prediction with temperature break prediction and causal discovery. All results were obtained on the University of the Western Cape HPC cluster using SLURM job scheduling. Timing results were obtained under controlled conditions, allocating eight CPU cores and 16GB RAM to match a standard PC configuration, while all other experiments utilized resources as required. Local development was carried out on an Asus VivoBook (Intel Core i7, 16GB RAM, Ubuntu Linux) using Visual Studio Code and Jupyter Notebooks.

#### 4.2.1 Temperature prediction baselines

Three baselines were included: persistence (${\hat{T}}_{t+1}=T_{t}$), a three-step recent mean, and an autoregressive model of order two, AR(2), with the lag order set from the Partial Autocorrelation Function (PACF) (Pillai et al., 2025). AR(2) coefficients were estimated on the training partition and applied one step ahead to observed history, matching the forecast horizon of the evaluated models.

#### 4.2.2 Temperature prediction ML models optimization

The new model architectures and the models used in the Conference Paper (CP) presented and published (MLP, ELM, XGB, and tGRU) for apples' cold room temperature prediction from Taguta et al. (2025) were all trained and optimized on the CP dataset for fair comparison. New models were optimized using random search (Taguta et al., 2026a; Konovalenko et al., 2021). A total of 200 trials were used for the model architectures due to their larger search space, while 100 trials were used for tGRU. Feature selection and hyperparameter optimization used the training partition exclusively, with 20% held out for validation, with the test set held out until final evaluation. This implies that all models evaluated were trained with the test set held out entirely, used only for final evaluation. The CP models kept their parameters, including data splitting strategies, features, and sequence length. The new model architectures used a sequence length of two, informed by PACF (Figure 1), and three splits (75% train / 25% test) used for ELM (Taguta et al., 2025) for fair comparison. The LSGR in this study has different layers and hyperparameters (Taguta et al., 2026a). It was optimized, trained, and evaluated using scaled features and the target (Section 4.1); thus, it has been reconfigured. More details on the parameter space and the optimal parameters are provided in Supplementary Tables S1, S2, respectively. The new architectures used feature group H shown in Supplementary Table S3 just like CP models.

**Figure 1:**
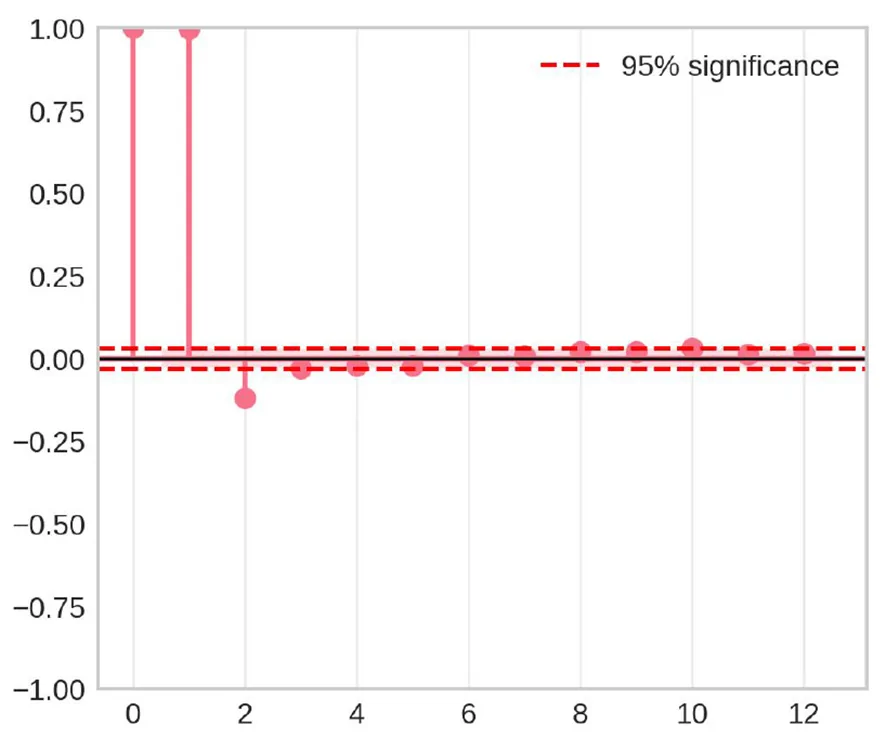
Partial autocorrelation of temperature; dashed lines mark 95% significance bounds.

#### 4.2.3 Temperature break prediction

Temperature break prediction involves not the value, but the classes, "break" or "no break," in which those values belong, based on safety-based regulated apples' cold room thresholds in South Africa. TBP is thus not performed by a separately trained classifier, as predicted temperatures are compared against the acceptable range based on Equation 19. Classification performance therefore depends on where predictions fall relative to the threshold boundaries rather than on average error alone. The temperature-break comparison is restricted to recurrent architectures. ELM and MLP are feed-forward networks, and XGB is tree-based; all three are reported as regression baselines for the dataset sensitivity analysis. The tGRU is included as a shallow recurrent baseline. The proposed models (LSGR, sLSTM, bGRU, and bLSTM) were re-optimized on the EDS for temperature prediction using the same parameter space (Supplementary Table S1), splitting strategy and windowing (Section 4.2.2), except that tGRU adopted the same splitting and windowing for fair comparison. The resulting continuous predictions were subsequently classified for temperature break prediction. Each model was executed 20 times using seeds 0–19 to ensure repeatability of results.

### 4.3 Sensitivity analysis

Models' sensitivity to datasets was investigated. Feature combinations, sequence length and number of splits (n_splits) were optimized in that order. For sensitivity analysis of sequence lengths, features, and cross-validation splits, model selection prioritized generalizability over absolute accuracy, with relative *R*^2^ stability under dataset transitions serving as the primary indicator of robustness across experimental conditions. The framework required candidates to demonstrate: (1) Superior adaptability to data distribution shifts, (2) Architectural transparency for unambiguous attribution of hyperparameter effects, and (3) Baseline-agnostic resilience to ensure consistent performance from varied starting conditions. Iterations or looping through the parameters being optimized were done during analysis, while the seeds were set (numpy, tensor, and random) to 0 for every iteration for generalization across iterations.

#### 4.3.1 ML models' dataset sensitivity: smaller vs. extended datasets

A comparative analysis was conducted to assess model generalizability across datasets of varying sizes and characteristics for temperature prediction. Specifically, the previously established models (Taguta et al., 2025) and the newly developed model architectures (Section 4.2.2 for optimization) were evaluated using both the original dataset and the extended dataset (Section 4.1). Normalised mean squared error (NMSE) and normalised mean absolute error (NMAE) were calculated on Min–Max scaled data (0–1) (Henderi et al., 2021) as shown in Equations 32, 33, respectively, so they will be lower than mean squared error (MSE) and mean absolute error (MAE) calculated on data that has not been unscaled. Thus, *R*^2^, NMSE, and NMAE were the evaluation metrics.

#### 4.3.2 Feature combinations

There is a strong need to find the best feature combinations as these affect ML models' prediction accuracy (Ayanoglu and Uysal, 2023; Zou et al., 2023; Meng et al., 2024). Feature groups, seen in Supplementary Table S3, were created based on domain knowledge and mutual information scores calculated using sklearn's mutual_info_regression function for each feature against temperature, the target (Huang et al., 2024). Figure 2 shows the scores. The partial autocorrelation function (PACF) is commonly used in time series forecasting to determine the number of past lags necessary for prediction. Thus, the PACF from Python's statsmodels module was used to determine the number of past temperature lags that directly influence its current value (Pillai et al., 2025), given that Temperature_Lagged was identified as the most influential feature (Figure 2). Figure 1 shows that only the past two lags are significant; hence, a sequence length of 2 was adopted to find optimum feature combinations. The 75%/25% train/test splitting strategy (3 n_splits) was used for baseline (Taguta et al., 2025).

**Figure 2:**
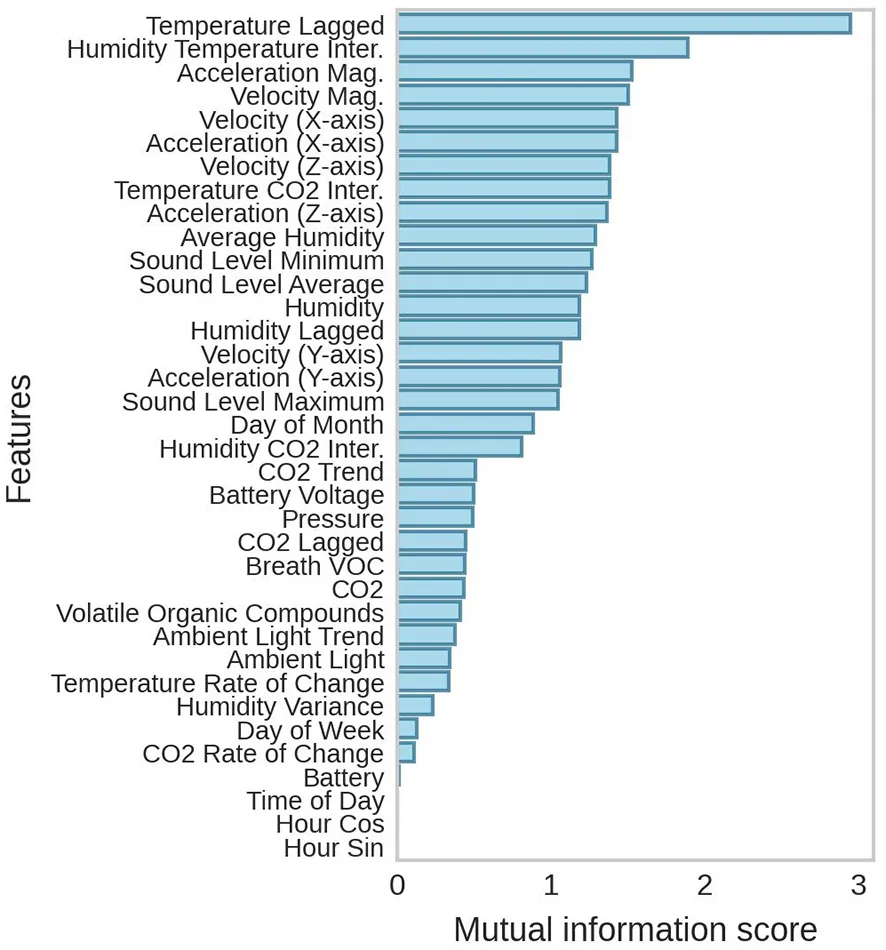
Mutual information scores between each feature and temperature, sorted descending.

#### 4.3.3 Sequence length

When preparing time-series data for time-series analysis models, it is critical to maintain proper chronological ordering of observations to preserve the temporal dependencies that the model aims to capture. Equally important is determining the optimal sequence length, that is, the number of past time steps included in each input sample, as this directly impacts the model's ability to learn relevant patterns and make accurate predictions (Machacuay et al., 2024). The authors considered sequence lengths of 2–19, 24 h. The minimum of 2 h was motivated by the PACF results for temperature lags (Figure 1), while the maximum considered the whole past day as reasonable for cold chain temperature prediction. The optimal feature group from the optimization process explained in Section 4.3.2 and a 75%/25% train/test split strategy (3 n_splits) were used.

#### 4.3.4 Splitting strategies

The splitting strategy critically impacts ML models' performance. Insufficient training data can cause underfitting, while an inadequate test set fails to reliably detect overfitting and yields unstable generalization estimates. Improper splits thus risk models that perform poorly on new data, hence the need for optimization. The optimal features and target, temperature, were split using scikit-learn's TimeSeriesSplit (Huang et al., 2024) to maintain the temporal dependencies, growing training sets with each iteration while keeping test sets looking forward in time. This is important because with time-series data, models have to be trained on past observations and validated on future observations, not randomly. To create a TimeSeriesSplit object, n_splits is required to determine how many splits or folds to create in the time-series data for cross-validation (Duarte et al., 2024). For this investigation, 2–20 splits were considered. The optimal sequence length and features found in Section 4.3.3 and Section 4.3.2, respectively, were used.

#### 4.3.5 Temperature prediction vs. temperature break prediction using EDS-optimized DL models

As explained in Section 3.1.2, regression results were converted to classification. The tGRU, LSGR, sLSTM, bGRU, and bLSTM regression and classification performances across both tasks were compared for consistency.

### 4.4 Explainability

At each time step, the trained model produces a temperature prediction, SHAP attributions are computed for the current input sequence, and causal inference is applied to a sliding window of recent observations. All three therefore operate per iteration rather than as batch analyses of the complete series. SHAP values were computed using shap.GradientExplainer (expected gradients method). PCMCI and LPCMCI were compared, with a lag-1 linkage (lagged causal discovery), representing the influence from the most recent past observation under investigation, simulating real-world real-time prediction systems where the most immediate past value would be the cause of an upcoming temperature, as predicted. The PC-based algorithms were implemented using Python's tigramite 5.2.8.2 package (Python Software Foundation, https://www.python.org). Based on the PACF values shown in Figure 1, a sequence length or lag order of 2 (tau_max) was used during LPCMCI and PCMCI comparison, with a pc_alpha of 0.05. The tau_max parameter, controlling the maximum causal look-back in time steps, was optimized over the range 1–3. The significance level (α) was varied across 0.05 and 0.1. At each prediction step, the most recent observation of the input sequence was retained, yielding one row per prediction in the series passed to the causal algorithm. Window length, the number of past observations available to the causal discovery algorithm, was varied over 10, 50, and 100 readings. These correspond to approximately 10 hours, 2 days, and 4 days of preceding history. The output matrices from the causal discovery process are first examined for infinite values to resolve numerical instability brought on by flat or nearly constant sections in a time-series window. A noise injection process is done across the series in the window before rerunning the analysis if any ∞ entries are found in either the *p*-value matrix or the coefficient matrix. The adjustment is carried out by comparing each point with its predecessor over the length of the series. When the absolute difference between them falls below a relative tolerance of 1 × 10^−12^, the point is considered stuck, and only runs of at least three consecutive stuck points are treated as flat. To break the flatness while maintaining the underlying signal, those points are next subjected to small perturbations derived from a zero-mean Gaussian distribution (σ = 1 × 10^−8^ of the standard deviation of the series itself). A rectified copy of the original series is returned, and the causal discovery process is repeated using the updated data, leaving the original series unaltered.

### 4.5 Evaluation metrics

The metrics used to evaluate ML models' temperature and temperature break performances, as well as explainability analysis, are presented in this section. Regression and break-prediction comparisons used a paired Wilcoxon signed-rank test (*W*-test) on metrics matched by seed (*n* = 20, seeds 0–19 per model), with Holm-Bonferroni (Holm) correction within each metric.

#### 4.5.1 TP metrics

The *R*^2^, MAE and MSE were used for evaluating the temperature predictive performance of the ML models. The closer the MAE and MSE are to 0 and the closer the *R*^2^ approaches 1, the better the performance (Jiang et al., 2023; Guo et al., 2024). Absolute error metrics such as MAE and MSE tend to increase when the target values are larger, even when relative predictive performance is similar or better. Normalizing these errors by the target-value range enables a fair comparison between the old and extended datasets, despite their different temperature ranges. Accordingly, the normalized mean squared error (*NMSE*) and normalized mean absolute error (*NMAE*) are computed as follows:


$$\begin{array}{l} \text{NMSE}=\frac{\text{MSE}}{{\left(y_{\max}-y_{\min}\right)}^{2}},and \end{array}$$ (32)

$$\begin{array}{l} \text{NMAE}=\frac{\text{MAE}}{y_{\max}-y_{\min}}, \end{array}$$ (33)

where y_*min*_ and y_*max*_ are the minimum and maximum target values, in this case, cold room internal temperature, respectively.

#### 4.5.2 TBP metrics

Precision, Recall, F1-Measure, and Accuracy were used for evaluating models' classification performance against the binary values created from actual test data. The key metrics for model ranking were Precision, Recall, and F1-Measure. The closer these are to 1, the better. Accuracy: quick global sanity check, but ignored for ranking because class imbalance can inflate it; Precision: keeps costly false alarms low (unnecessary interventions); Recall: ensures nearly every real break is caught; missing one is far costlier than a false alert; and F1: single harmonic-mean score that balances precision and recall on imbalanced data (Mangini et al., 2020; Xie et al., 2025; Swain and Jenamani, 2023a). Confusion matrices were also used to determine how each model predicted classes against the correct classes in the test data for TBP. The fewer the false positives and negatives, the better the model's performance (Mangini et al., 2020). These measures score individual time steps, so episode-level measures were used alongside them. Break episodes were identified as maximal runs of consecutive time steps outside the acceptable range. Event recall is the proportion of episodes flagged at any point during them; false alarms are reported per day of monitoring, and anticipation is the number of episodes flagged before onset, searched over the 6 h preceding each. Where episodes were flagged before onset, inspection confirmed the flag persisted through to onset in each case. Episodes beginning at the start of the test period are excluded from the anticipation count, as no preceding window exists.

#### 4.5.3 Time metrics

The time it took to train each model using the EDS was measured using Python's time module's *time* function, while for causal latency, the *perf*_*counter* function was used. The difference between timestamps before and after the tasks (training or causal discovery) was computed.

#### 4.5.4 Feature prioritization and statistical analysis

The rankings of out-of-range features using SHAP, LPCMCI causality and a threshold-based approach when a temperature break was predicted were compared to determine if feature deviation magnitudes correlate with causality while comparing how causation relates to correlation (Causal vs SHAP). To quantify agreements, the following were calculated: (1) Spearman rank correlation coefficients, (2) Top-K overlap scores for the most important features, and (3) 95% confidence intervals using the Wilson method. These comparisons served to validate the LPCMCI, which was also validated by inspecting the results in light of domain knowledge. The agreement between causal graphs recovered at different window lengths is quantified by containment (cont.), defined as:


$$\begin{array}{l} C\left(A,B\right)=\frac{\left|A\cap B\right|}{\left|A\right|}, \end{array}$$ (34)

where *A* and *B* are the sets of (feature, position) links recovered at the shorter and longer window lengths. The measure is asymmetric, as a longer window is expected to recover additional links given its larger sample.

## 5 Results and discussions

This section presents and discusses the study's results, beginning with an analysis of ML model sensitivity to dataset variations, from which one model is selected for further investigation of feature combinations, sequence length, and splitting strategies. This is followed by an examination of temperature break predictions, where the best-performing model's outputs are integrated with explainability approaches for deeper analysis.

### 5.1 Sensitivity analysis

The results and discussions for the ML models' sensitivity to datasets (ODS vs. EDS) and selected models' sensitivity to feature groups, splitting strategies and sequence length are covered below.

#### 5.1.1 Datasets sensitivity for TP

Using models optimized and trained on ODS, Table 1 shows that the CP models performed moderately on the ODS, except that the ELM was outstanding across all metrics (*R*^2^: 0.9835, MAE: 0.0709, and MSE: 0.0299). Unlike in Taguta et al. (2025), where optimistic results were reported for MLP and tGRU since they used test data for early stopping, with MLP and tGRU performing better, testing on unseen data in this study reduced their performance, even much more for tGRU (*R*^2^: 0.8018 vs. 0.9720, MAE: 0.4030 vs. 0.1188 and 0.6063 vs. 0.0856). This further confirms the earlier findings in the previous study that more data is required for training GRU for improved performance. DNNs performed very well, especially the LSGR, highlighting the inherent benefits of combining the lightweight GRU with the LSTM's capacity for long-term dependencies (Cho et al., 2014; Hochreiter and Schmidhuber, 1997). The poor performance of stacked LSTM layers confirms the lack of the aforementioned LSTM and GRU stacking performance benefits. Despite being the least performing, sLSTM predictions deviate by approximately 0.6°C.

**Table 1:** ML models TP performance on old and extended datasets.

Model/ Metric	tGRU	MLP	ELM	XGB	LSGR	sLSTM	bGRU	bLSTM
Predictions with old dataset								
*R^2^*	0.8018	0.9454	**0.9835**	0.9733	0.9791	0.7361	0.9375	0.9253
MAE	0.4030	0.2141	**0.0709**	0.1138	0.1027	0.6327	0.2497	0.2054
MSE	0.6063	0.1603	**0.0299**	0.0782	0.0383	0.4829	0.1143	0.1367
NMAE	0.0707	0.0376	**0.0124**	0.02	0.0180	0.1110	0.0438	0.0360
NMSE	0.0187	0.0049	**0.0009**	0.0024	0.0012	0.0149	0.0035	0.0042
Predictions with extended dataset								
*R^2^*	0.7391	0.9753	**0.9955**	0.9405	0.9851	0.9566	0.9766	0.9882
MAE	2.2811	0.5311	**0.2202**	1.0048	0.8087	1.9303	1.0892	0.6264
MSE	7.2439	0.9354	**0.5839**	2.2503	1.9432	5.6530	3.0522	1.5339
NMAE	0.0750	0.0175	**0.0072**	0.0331	0.0266	0.0635	0.0358	0.0206
NMSE	0.0078	0.0010	**0.0006**	0.0024	0.0021	0.0061	0.0033	0.0017

Bold is better.

The ELM did very well on the EDS, topping the performance as depicted in Table 1. The ELM is the overall best across datasets, explained by fast learning and good generalizability (Huang et al., 2006). Though MLP had good MAE and MSE performance, CP models were generally outperformed by the DNN models, especially with respect to *R*^2^, where most DNNs achieved an *R*^2^ above that of the second-placed CP model, MLP (0.9753); thus, the DNNs generalize better across datasets. This is evidenced by performance gains of DNNs across datasets. *R*^2^ changed by −7.8% to +30% across the eight models, with several DNNs improving the most. The sLSTM improved the most, while two CP models (XGB, tGRU) degraded, with tGRU degrading the most. Contrarily, MLP gained the most NMSE (79.5%), followed by some DNNs which moderately improved, except for LSGR, which degraded by 75%, while most CP models generally improved performance. This indicates that the LSGR model was affected by sharp spikes in the extended dataset. Despite the huge performance loss, the LSGR ranks 4^th^, doing better than most models, hence resilient. A similar trend follows the NMAE metric change, though it ranged from −53.5% to +65.5%, with XGB having the most performance loss. These results indicate that DNNs gained performance across datasets while standard ML models like XGB and tGRU lost performance. This indicates that more data improves DNNs' performance and implies that complex models like DNNs can explain the data more when more data becomes available, and their performance improves significantly, as expected in Taguta et al. (2025). This is also due to the inherent capability of LSTMs and GRUs to capture complex, non-linear patterns (Ghoroghi et al., 2023; Hoang et al., 2021). The inclusion of the LSGR model can thus be justified among new architectures, as it is the second best across all metrics on ODS and is the second best DNN on EDS, and better than most CP and new models. It can be argued that it is the most consistent DNN since it is the only recurrent model ranked in the top four on both datasets, holding its position under the dataset transition, which meets the *R*^2^ stability criterion. Its recurrent form also suits sequence-length sensitivity. ELM, though highest-ranked, is a closed-form single-layer model and serves only as a reference. Overall, it can be argued that DNN models performed very well on the extended dataset, even though they were optimized on a smaller dataset, hence good generalization to the shifted operating conditions of the extended dataset. This is a major contribution of this study, enabling the assessment of ML models' generalizability using a richer, diverse dataset, and demonstrating that DNN models may be deployed across different cold chain environments with consistently good performance.

#### 5.1.2 Feature combinations

The groups of features can be seen in Supplementary Table S3. As shown in Figure 3A, feature group I has the lowest MAE (0.0190), while group A, shown in Figure 3B, has the highest *R*^2^ (0.9899). This implies the model requires more key features, including temperature (lags), humidity, CO_2_, and ambient light, while for a good fit, it relies on a group comprising temperature (target) related features. The presence of A in the top-2 shows that features linked to the target are more important for temperature prediction, as supported by MIS (Figure 2) and PACF (Figure 1). Internal temperature was also found important in Zou et al. (2023), while other features produced good results in past studies (Taguta et al., 2025; Loisel et al., 2022; Haider et al., 2024; Taguta et al., 2026a). This implies that group I has features that are key for accurate temperature prediction in the FFVs cold chain.

**Figure 3:**
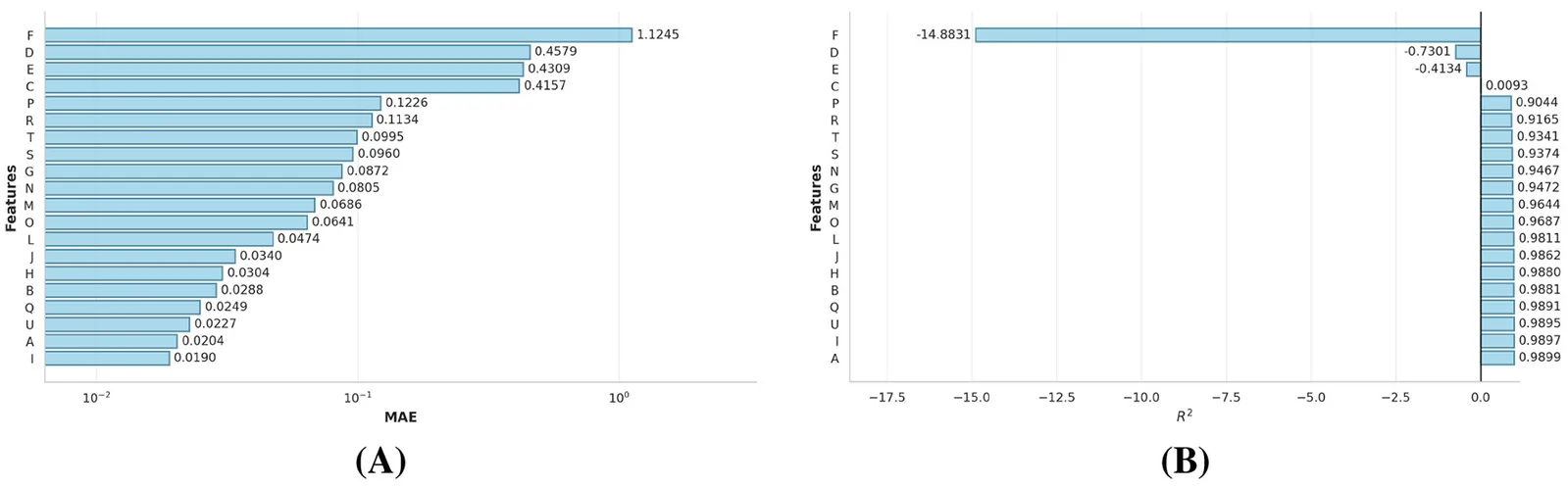
Test MAE (°C) and *R*^2^ across feature groups, sorted best-to-worst. **(A)** MAE. **(B)** *R*^2^.

Feature group U is a strong candidate as well. By attaining better performance (MAE: 0.0227, *R*^2^: 0.9895), it means that velocity-related features are not important for the model architecture under study. Q also produced good performance (MAE: 0.0249, *R*^2^: 0.9891) over many feature groups, signifying the importance of past temperature lags to temperature prediction, concurring with MIS and PACF, though the single feature is not the only feature required for accurate predictions since its performance is poor compared with groups I and U. Feature groups B-L produced very competitive results (*R*^2^: 0.9881–0.9811, MAE: 0.0288–0.0474), highlighting that these are potential optimal features for the LSGR. This is mainly because they contain the most key features in group I, except that group B lacks past temperature lags, and group H has extra motion-related features like groups J and L, which have high MI scores (Figure 2). Groups O-P also produced decent performance, with *R*^2^ above 0.9. Their lower results may be explained by the inclusion of many features in most of these groups, which may have introduced noise. Only groups C, E, D, and F, which comprise light, motion, CO_2_, and more motion features, respectively, resulted in lower *R*^2^ and higher MAE, thus very poor performance. This is because the groups exclude other key features. Overall, group I was selected as the optimal feature group since it produced temperature predictions which are closer to the target.

#### 5.1.3 Sequence length

As shown in Figures 4A, B, the sequence length of two has the lowest MAE (0.019) while 19 has the highest *R*^2^ (0.9913), respectively. These results signify that a lower sequence length helps the model to predict the temperature more accurately but at a fit cost, while a longer window results in better fit but poor point-to-point accuracy. With a sequence length of 19 also producing the second best MAE, it can be argued that longer histories remain informative rather than introducing noise. Interestingly, the transition from 2 to 19 increased the MAE by a bigger margin (5.5%) compared to the insignificant *R*^2^ performance loss (0.2%). Importantly, the transition increased the training time by 530% from a length of 2 (22.8s) to 19 (143.8s), thus 2 has lower training latency, which may suit adaptive learning for real-time applications. This means a sequence length of 2 is optimum as it results in lower absolute errors and still a comparably good fit. This is mainly because temperature is strongly autocorrelated at short lags (Figure 1), with Temperature_Lagged the highest-ranked feature by mutual information (Figure 2). Most studied lengths produced an *R*^2^ above 0.9 and lower MAE, indicating that the 2–19 past hours remain useful for cold room temperature prediction. However, a day's history produced lower *R*^2^, indicating that it introduces noise to the model. It may thus be argued that a larger sequence length does not guarantee better performance, disagreeing with Xie et al. (2025). As sequence length increased, training time increased (*r* = 0.681), thus with longer sequence lengths generally comes more training time, agreeing with the same study. Therefore, the sequence length of two is optimum for temperature prediction in the FFVs cold chain.

**Figure 4:**
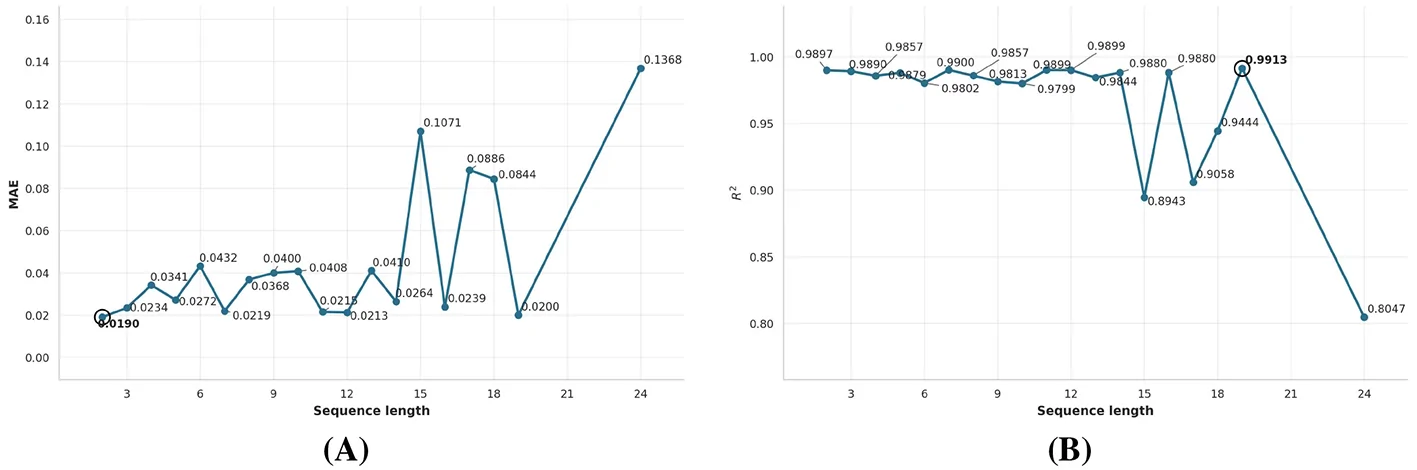
Test MAE (°C) and *R*^2^ across sequence lengths; the circled point marks the best value. **(A)** MAE. **(B)** *R*^2^.

#### 5.1.4 Splitting strategies

To discover how the model is sensitive to the number of splits, 2–20 were utilized, with Figures 5A, B showing achieved MAE and *R*^2^, respectively. Test *R*^2^ was stable between two and six splits (0.9897–0.9683), with three splits producing the best goodness of fit, then became erratic, dropping to 0.0706 at 13 splits and −0.4826 at 20 splits, though training *R*^2^ stayed above 0.96 throughout. MAE gave the opposite order, favoring the higher splits; the lowest MAE (0.0106) was at 12 splits, whose test partition is only 7.64% of the data, against 25% at 3 splits. The implied test variance, MSE/(1 – *R*^2^), fell as splits increased, meaning *R*^2^ is not comparable across the configurations. A three-split strategy was selected, giving the best *R*^2^ in the stable region (0.9897) while keeping a larger test set. As splits increase, each test partition covers a shorter and more homogeneous window, which shrinks the variance term in the denominator of *R*^2^. This means that a model with only a modest absolute error can still record a negative *R*^2^, as seen at 20 splits. The low MAE at the higher splits is similarly explained by the narrower evaluation window and the greater sampling uncertainty of an estimate from few observations. Since training *R*^2^ was stable across the whole range, the model was not data-limited at any split count, so the choice is one of measurement reliability rather than data sufficiency.

**Figure 5:**
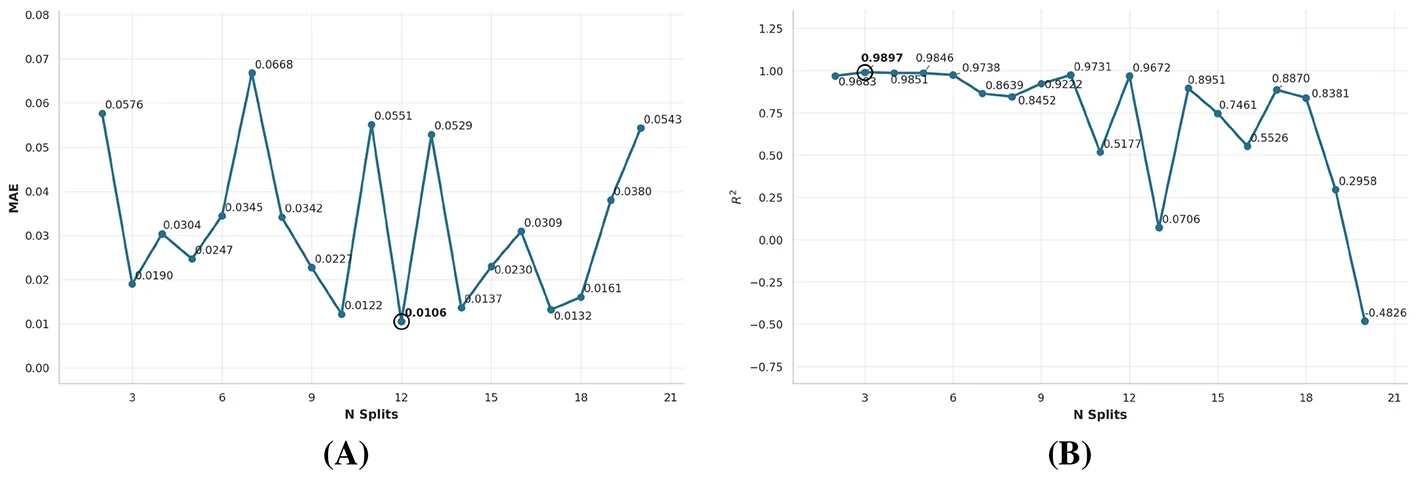
Test MAE (°C) and *R*^2^ across number of splits; the circled point marks the best value. **(A)** MAE. **(B)** *R*^2^.

#### 5.1.5 Application of optimal features

Using the optimal parameters (sequence length: 2, splits: 3 and features group: (I) and a random seed of 0, the LSGR performed better (MSE: 1.3423, MAE: 0.5037 °C, *R*^2^: 0.9897, Precision: 0.9887, Recall: 0.9957, F1: 0.9922, and Accuracy: 0.9889) compared to the LSGR architecture optimized on EDS, using feature group H (Section 4.2.2) and random seed of 0, which is part of 0–19 seeds results reported in Section 5.2.1 (MSE: 1.5632, MAE: 0.8051, *R*^2^: 0.9880, Precision: 0.8886, Recall: 1.0000, F1: 0.9410, and Accuracy: 0.9111). The optimal parameters thus improved the MSE by 14%, MAE by 37%, Precision by 11%, and Accuracy by 9%. The significant increase in regression and break prediction performance can therefore not go unnoticed, especially the 90.91% reduction in predicted false alarms (false positives or breaks), from 88 to 8, accompanied by only a slight increase in missed breaks (false negatives), from 0 to 3. The comprehensive parameter optimization for FFVs cold chains is a major milestone, providing a strong foundation on the minimum sensors or features (cost-effective), the number of past readings (affecting model complexity), and the splitting strategy (affecting performance) required for ML-accurate temperature modeling for increased cold chain integrity.

### 5.2 Temperature and Temperature Break Predictions

The use of DNN models for temperature break prediction is justified by their increase (sLSTM, bGRU, and bLSTM) and consistency (LSGR) in performance on an EDS as shown in Table 1, indicating a need for re-optimization. However, the tGRU was used as a baseline shallow model for comparison with DNNs. After optimization on the EDS, the chosen models had their TP results improved significantly as obtained using *seed* = 0, except for the bLSTM, whose MAE, MSE, and *R*^2^ performances decreased by 50.7%, 41%, and 0.5%, respectively. The performance gains indicate the importance of optimizing the models on the bigger, richer, and more diverse dataset. The results for both TP and TBP are shown in Figures 6A, B, respectively, where the best model's bar is striped through. Tables 2, 3 show the paired Wilcoxon signed-rank comparisons for both tasks over 20 seeds, with Holm–Bonferroni correction.

**Table 2:** Paired *W*-test comparisons of TP performance against LSGR across 20 seeds.

Metric	vs.	Mean diff.	*p*	*p*_Holm_
MSE	bGRU	–0.415	0.330	0.461
MSE	bLSTM	–0.520	0.231	0.461
MSE	sLSTM	–2.452	< 0.001	< 0.001
MSE	tGRU	–6.243	< 0.001	< 0.001
MAE	bGRU	–0.066	0.756	1.000
MAE	bLSTM	–0.092	0.648	1.000
MAE	sLSTM	–0.617	< 0.001	< 0.001
MAE	tGRU	–1.140	< 0.001	< 0.001
*R* ^2^	bGRU	+0.003	0.330	0.461
*R* ^2^	bLSTM	+0.004	0.231	0.461
*R* ^2^	sLSTM	+0.019	< 0.001	< 0.001
*R* ^2^	tGRU	+0.048	< 0.001	< 0.001

+ differences favor LSGR for *R*^2^ and − differences favor LSGR for MSE and MAE. Holm correction applied within each metric.

**Figure 6:**
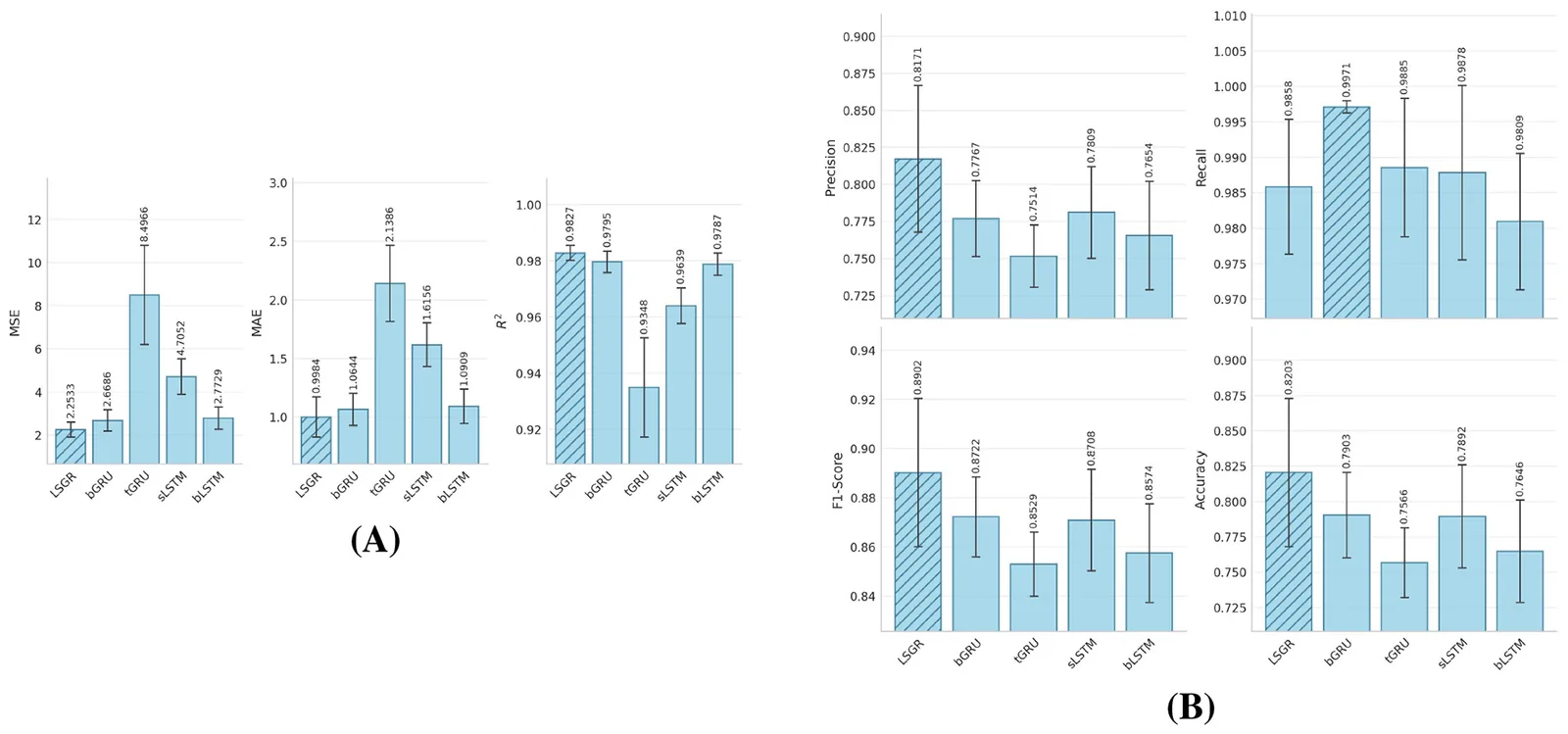
Model performance with 95% confidence intervals over 20 seeds. **(A)** TP [MSE, MAE (°C); *R*^2^]. **(B)** TBP (precision, recall, F1, and accuracy).

**Table 3:** Paired *W*-test comparisons of TBP performance against LSGR across 20 seeds.

Metric	vs.	Mean diff.	*p*	*p*_Holm_
Precision	bGRU	+0.040	0.154	0.461
Precision	bLSTM	+0.052	0.277	0.461
Precision	sLSTM	+0.036	0.218	0.461
Precision	tGRU	+0.066	0.022	0.087
Recall	bGRU	–0.011	0.047	0.187
Recall	bLSTM	+0.005	0.586	1.000
Recall	sLSTM	–0.002	0.394	1.000
Recall	tGRU	–0.003	0.472	1.000
F1-Score	bGRU	+0.018	0.261	0.522
F1-Score	bLSTM	+0.033	0.154	0.461
F1-Score	sLSTM	+0.019	0.351	0.522
F1-Score	tGRU	+0.037	0.036	0.146
Accuracy	bGRU	+0.030	0.330	0.660
Accuracy	bLSTM	+0.056	0.154	0.461
Accuracy	sLSTM	+0.031	0.355	0.660
Accuracy	tGRU	+0.064	0.040	0.161

+ differences favor LSGR. Holm correction applied within each metric.

#### 5.2.1 Temperature prediction

As can be seen in Figure 6A, DNNs performed very well, with the LSGR achieving the lowest average MSE (2.2533) and MAE (0.9984) and the highest *R*^2^ (0.9827), making it the best DNN. The good LSGR performance may be attributed to the gating in the LSTM and GRU units, which allows them to capture the temporal dependencies in the data (Ghoroghi et al., 2023; Hoang et al., 2021). The bidirectional complexity of the bGRU and bLSTM may have contributed to their poorer performance compared to the LSGR, as they have more parameters and may require even more data with less noise (Hoang et al., 2021). The tGRU has the poorest performance, which may be attributed to a single unidirectional layer having too little capacity to learn these dependencies. This implies that performance does not simply increase with complexity, and that the LSGR sits at a level of complexity that suits this FFVs cold chain data and temperature prediction task. Statistically, there is no significant difference (*p*>0.05, *p*_Holm_>0.05) between LSGR and bidirectional models, as shown in Table 2. The Table also indicates that LSGR significantly and practically outperforms sLSTM and tGRU, confirming the earlier findings. However, LSGR was outperformed by the baselines: Persistence (MAE: 0.1950, MSE: 0.4974, *R*^2^: 0.9962), Recent mean (MAE: 0.2828, MSE: 0.8427, *R*^2^: 0.9935, AR(2) (MAE: 0.2019, MSE: 0.5024, *R*^2^: 0.9961). Lagged temperature, the key feature by MIS (Figure 2), is highly autocorrelated at lag 2 (Figure 1) due to thermal inertia, so one-step prediction approximates a random walk, which the baselines capture directly. This is supported by ELM, the simplest learned model, outperforming the deep architectures (Table 1) and the recent-mean baseline, while itself being only slightly outperformed by persistence and AR(2). It is further supported by the sensitivity analysis (Section 5.1.2), where LSGR achieved its lowest MAE with multi-feature groups rather than lagged temperature alone. Variables providing such structure, including door state, compressor status, and external ambient temperature, were not instrumented. Overall, the LSGR remains the best ML model for temperature prediction in FFVs cold chains.

In terms of training times, Table 4 shows that the average duration increases in the tGRU, LSGR, bLSTM, sLSTM, and bGRU order. It should be noted that these are the training times at each model's optimized configuration, so they reflect the tuned hyperparameters as well as the architecture. The tGRU is the fastest to train, as its single layer has the fewest parameters to update per epoch, though this comes at the expense of predictive performance. The LSGR is the second fastest, training 37% faster than the sLSTM. This is because the LSTM-GRU combination is less costly per epoch than two stacked LSTM layers, since the GRU layer has fewer gates (Cho et al., 2014; Ghoroghi et al., 2023), while still retaining enough memory to converge well. The bLSTM and sLSTM have lower training times, as processing each sequence in both directions and stacking a second layer both increase the computation of a single layer. The bGRU is the slowest, which is likely due to its smaller batch size of 16 compared to 32 for the bLSTM and sLSTM, as this increases the number of gradient updates per epoch.

**Table 4:** Models' training times (mean and std for 20 seeded runs).

Model	bGRU	LSGR	tGRU	bLSTM	sLSTM
Train time (s)	37.8 ± 5.8	20.3 ± 1.9	**13.7** ± 3.4	29.7 ± 5.4	32.1 ± 4.9

Bold is better.

#### 5.2.2 Temperature break prediction

The LSGR is the best model for TBP in terms of Precision (0.8171), F1-Score (0.8902), and Accuracy (0.8203), as shown in Figure 6B and supported in Table 5, where the model has the highest average true negatives (120) and the lowest false positives (167.9). This may reflect the GRU layer's smaller parameter count (Cho et al., 2014), which limits overfitting to spurious positive-class patterns, combined with the LSTM layer's capacity for long-term dependencies (Hochreiter and Schmidhuber, 1997; Hoang et al., 2021). However, Table 3 shows that much of the performance difference is not significant, except for tGRU's Precision but not after correction. The LSGR has the highest Recall (0.9858) among its metrics but is lower than most models, implying that the model forgets actual temperature breaks more than other DNNs. The bGRU has the best Recall (0.9971), but insignificant compared to LSGR and is second-best across other break prediction metrics, except for Precision, since the sLSTM was more precise (0.7809) than the bGRU. Despite not-so-good regression performance, the sLSTM performed very well in break prediction. This may be attributed to the long-term dependency learning of LSTMs (Hoang et al., 2021). This also implies that poor regression performance does not necessarily translate into poor classification performance: predictions that are slightly inaccurate, and therefore penalized by regression metrics, may still fall on the correct side of the break threshold and yield accurate classification, a significant finding of this study, providing a footing for accurate temperature break prediction and proactive cause identification as encouraged in Taguta et al. (2026b). The bLSTM ranks 4th while tGRU ranks 5th across all metrics. Despite the poor classification metrics, the tGRU has the second-best Recall (0.9885). It is more biased toward predicting temperature breaks at the expense of raising more false alarms (false breaks), as depicted also in Table 5. This also confirms the regression vs. classification performance finding. Given that most models achieved Precision, F1, and Accuracy below 0.9, it can be argued that the models still require more training and testing data to improve performance.

**Table 5:** Confusion matrix values (TN, true negative; FP, false positive; FN, false negative; TP, true positive).

Model	TN	FP	FN	TP
bGRU	82.5 ± 64.3	205.6 ± 64.3	**2.0** ±1.3	**700.0** ±1.3
bLSTM	68.3 ± 82.5	219.7 ± 82.5	13.4 ± 14.4	688.6 ± 14.4
LSGR	**120.0** ± 108.7	**167.9** ± 108.7	9.9 ± 14.3	692.0 ± 14.3
sLSTM	87.9 ± 72.8	200.1 ± 72.8	8.6 ± 18.4	693.5 ± 18.4
tGRU	55.1 ± 55.1	232.9 ± 55.1	8.1 ± 14.7	694.0 ± 14.7

Bold is better.

All models achieved an event recall of 1.00, flagging every break episode at some point (Table 6), which is expected given episode durations of 56–470 h. False alarms ranged from 4.07 to 5.65 per day, with LSGR raising the fewest. Of the two predictable events (the first begins at the start of the test period and has no preceding window), the DNNs flagged between 1.25 and 1.80 ahead of onset, with the flag sustained through to onset. No baseline flagged any event in advance. The differences between DNNs on anticipation are small relative to seed variation and are not interpreted as a ranking. The distinction from the baselines is categorical: persistence, recent mean, and AR(2) report only that temperature has already left the band, and cannot warn in advance by construction. This is the principal operational advantage of the learned models, notwithstanding their comparable regression accuracy. The false alarm rate of 4–5.7 per day remains high for deployment; requiring consecutive break predictions before raising an alarm would reduce it, but was not evaluated here. Therefore, statistically, there exists stiff competition between LSGR and other models, though LSGR emerges as the best model for temperature break prediction. Overall, the LSGR is the best model for both temperature prediction and temperature break prediction of the recurrent models assessed, as it has consistent performance across both tasks.

**Table 6:** Event-level and operational break-prediction metrics (means over 20 seeds with std in brackets).

Model	Event recall	False alarms/day	Events early
bGRU	1.00 (0.00)	4.98 (1.56)	1.60 (0.50)
bLSTM	1.00 (0.00)	5.32 (2.00)	1.60 (0.60)
LSGR	1.00 (0.00)	4.07 (2.64)	1.25 (0.72)
sLSTM	1.00 (0.00)	4.85 (1.76)	1.40 (0.60)
tGRU	1.00 (0.00)	5.65 (1.34)	1.80 (0.41)

### 5.3 Explainable deep learning

The integration of LSGR with features' causality and importance to predicted temperature for explaining the model's output, coupled with features' deviation magnitude for countermeasure scoring upon break prediction, is covered hereunder.

#### 5.3.1 PCMCI vs. LPCMCI

When comparing each of the measured features' (Temperature_Lagged, Humidity, Ambient Light, and CO_2_) causality based on the two algorithms, Figure 7A shows that both methods agree on the main causal driver of temperature changes, that is, Temperature_Lagged (39). This implies that past temperatures are highly causal to predicted temperatures. Second, they concur that CO_2_ (15) also drives temperature changes while ambient light does not. These views are also supported in Figure 7B. However, PCMCI has more independent discoveries on each of the parameters, totalling 549 for all features, than LPCMCI (56), especially for humidity, which was not found causal by LPCMCI. Of the causal links, LPCMCI removes more connections, that is, 89.8%. PCMCI records instances where the graph shows the target to variable as causal, yet LPCMCI shows uncertainty. In a few cases, there were surely variable-to-target solid links for PCMCI and very few for LPCMCI on the same iteration, meaning most are surrounded by uncertainty as LPCMCI doubts most of the connections as target-to-variable and vice versa and a few bidirectional solids. The LPCMCI doubts are shown by the double uncertainty (o-o) and to-and-from connections ( < ->) and (o->), though PCMCI records them as causal because it cannot handle latent confounders, yet LPCMCI does not confirm such relationships (Runge et al., 2019b; Gerhardus and Runge, 2020). This implies that most PCMCI causal links are doubted by LPCMCI, thus have uncertainty, and maybe due to unmeasured parameters (latent confounders), a key contribution of this study for the FFVs cold chain. Hence, LPCMCI is the algorithm of choice, especially since many parameters may be unmeasured in cold rooms, for instance, O_2_ and product temperature, among others. The low detection rate, especially for LPCMCI (PCMCI: 55.9%, LPCMCI: 5.7% of 3,924 candidate links; 4 features × 981 analyzed windows), implies that there are other major causes of predicted temperature changes, and not just the measured parameters. These may be unmeasured parameters or the interactions of the four parameters.

**Figure 7:**
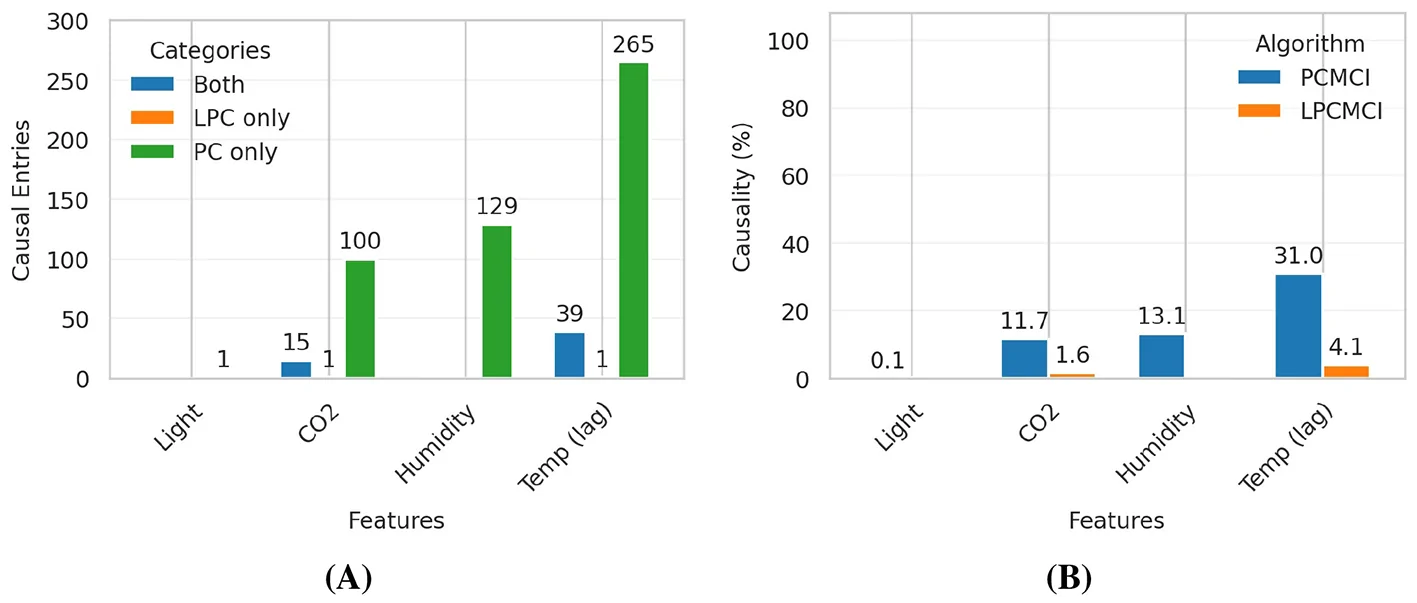
PCMCI vs. LPCMCI: **(A)** Causal feature agreement over 981 windows. **(B)** Feature-level causality comparison.

#### 5.3.2 LPCMCI robustness

##### 5.3.2.1 ParCorr vs. RobustParCorr

The LPCMCI requires conditional independence tests. ParCorr struggled to capture causal links compared to RobustParCorr for Temperature_Lagged (11 vs. 40), Humidity (1 vs. 0), CO_2_ (3 vs. 16) over 981 analyzed windows. RobustParCorr was selected for all subsequent LPCMCI analyses. It captured almost 273% more causal links than ParCorr, highlighting the importance of robust methods for climate causal discovery, where non-Gaussian characteristics such as seasonal cycles and extreme weather events bias standard correlation estimates. These systematic violations of Gaussian assumptions in climate data (Perron and Sura, 2013) make robust approaches essential for reliable driver identification. RobustParCorr addresses this by applying a non-paranormal transformation, which maps marginals to standard normal distributions via rank-based empirical distribution functions, thereby mitigating the effects of non-Gaussianity and outliers.

##### 5.3.2.2 LPCMCI parameter optimization

The causal graphs using LPCMCI revealed strong drivers of predicted temperature, including Temperature_Lagged and CO_2_. To assess the robustness of these findings to the choice of tau_max, a sensitivity analysis was conducted. The optimal tau_max, 2, was selected as the value producing the highest causal links (56) over tau_max of 1 (3) and tau_max of 3 (2). A significance threshold of 5% was used, as a 10% threshold increased causal links captured by 12.5% to 63, implying that the additional links are more likely false positives than true causal interactions. Interestingly, the two values have more common causal links (CO_2_: 14, Temperature_Lagged: 37), indicating that the links were not captured by chance. Features' causality varied across tau_max ∈{1, 2, 3}, suggesting the conclusions are sensitive to the choice of maximum lag parameter and not the significance threshold.

Causal discovery was repeated at window lengths of 10, 50, and 100 observations. Each pairwise comparison was restricted to positions available to both window lengths in that pair. As shown in Table 7A, the number of recovered links increased with window length for Temperature_Lagged. Both window lengths of 50 and 100 had increased causal links for all features compared to the window length of 10. This is consistent with the greater statistical power available at larger sample sizes. The table also shows that only temperature lag is the main causal driver of predicted temperature, consistent with PACF (Figure 1). Light was found not to be causal to predicted temperature under all studied windows, just like in Section 5.3.1, signifying that it is non-causal to predicted temperature in this investigation. Temperature_Lagged was the only feature recovered at every window length, with containment (Equation 32) between window lengths ranging from 0.60 to 0.68. Overall containment across all features ranged from 0.43 to 0.68 (Table 7B), indicating partial reproducibility of recovered links across window lengths. The strongest links show moderate stability from 10 to 100 past observations, while weaker links are more window-dependent. Median causal discovery latency increased as the window length increased (10: 61 ms, 50: 205 ms and 100: 364 ms). This implies that more causal links are recovered at longer window lengths, at an increased computational cost per iteration and a longer delay before the first output. The operational window is set to 10 observations. Causal inference is intended to inform response to an imminent break, for which recent conditions are the relevant context; a window of 100 observations spans over four days and delays the first causal output accordingly. The sensitivity analysis quantifies the cost of this choice: fewer links are recovered, and only the strongest links are reproduced consistently at longer window lengths. The causal findings are therefore presented as exploratory temporal links rather than established causal drivers.

Table 7Sensitivity of causal discovery to the analysis window.

**Table:** 

Feature	*w*_10_	*w*_50_	*w*_100_
(A)			
Temperature_Lagged	40	354	429
CO_2_	16	32	14
Humidity	–	12	11
Ambient Light	–	–	–
Total	56	398	454

**Table:** 

Comparison	From	|*A*|	Common	Cont.
(B)				
10 vs. 50	49	56	24	0.43
10 vs. 100	99	40	27	0.68
50 vs. 100	99	349	195	0.56

(A) Links recovered per feature at each window length.

(B) Pairwise agreement from the position in *From*, where *Common* is the subset of the shorter window's links also found at the longer one. Dashes indicate features not recovered.

#### 5.3.3 Parameter's causality analysis

Using the LPCMCI, Figure 8, in which darker colors represent poor performance for causal, *p*-value, and causality strength, shows that past temperature is the main driver of future temperature, as it has a higher average causality, followed by CO_2_. Humidity and ambient light remain not causal at all. Figures 7A, B also confirm the causality impact of these parameters. Thus, based on the four measured features, ambient light was not identified as a direct causal driver of the temperature changes in the dataset used in this study, which is key to guiding future studies on what to measure and what not to measure for accurate predictive temperature modeling in the FFV cold chain.

**Figure 8:**
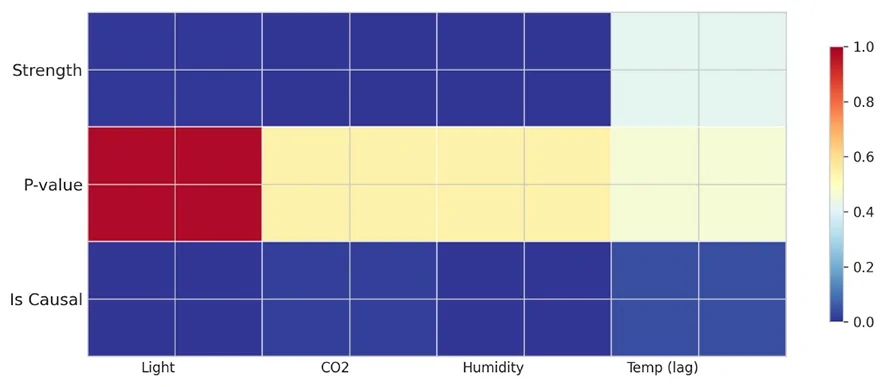
LPCMCI causal links to predicted temperature: causal status, link strength, and *p*-value per feature.

#### 5.3.4 Causality (LPCMCI) vs. importance (SHAP) for TP

As indicated in Figures 8, 9, Causality and SHAP agree that temperature lag is causal and important to predicted temperatures, thus requiring more priority for control to avoid or reduce temperature breaks. Figure 9 shows that lagged temperature is by far the most influential feature when predicting temperature, with a mean absolute SHAP value of 0.11004. CO_2_ ranks second, and humidity and ambient light contribute negligibly. Both approaches also agree that humidity and ambient light are neither important nor causal to predicted temperatures. While the SHAP analysis successfully identifies the key features that drive the model's predictions, it is crucial to note that these explanations are inherently associational. SHAP brilliantly quantifies how much each feature contributes to the model's output, but cannot distinguish whether a feature's influence is causal, confounded, or merely spurious. For instance, the model might correctly learn that an increase in current temperature is a strong predictor of temperature break, but from its perspective, this relationship is statistically indistinguishable from the (spurious) association between rooster crows and sunrise, both of which are patterns that aid prediction without revealing the underlying causal mechanism. Therefore, while SHAP provides invaluable insight into the model's behavior, it does not necessarily reflect the true physical causality of the system, as correlation is not causality (Ahmed et al., 2025; Docquier et al., 2024; Runge et al., 2023). The alignment of the two approaches on the most critical factors affecting predicted temperature (past temperature, CO_2_) is a huge milestone, informing (i) cold chain operators of the parameters to keep an eye on and (ii) ML modeling practitioners on which features matter the most, reducing sensing cost while improving ML performance. These are important for reducing FFVs' wastage, increasing food security, and helping stakeholders maintain the quality and safety of fresh produce throughout the cold chain.

**Figure 9:**
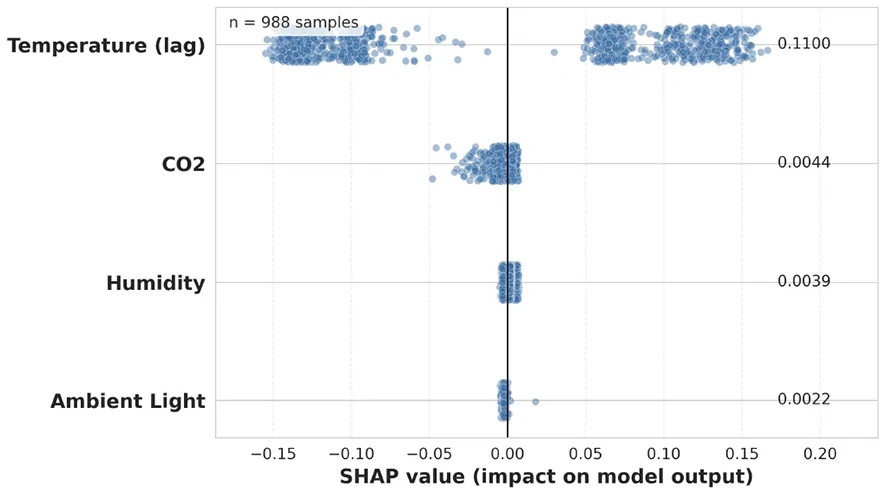
SHAP feature importance for TP.

#### 5.3.5 Temperature break and cause prediction

This section presents alerting and countermeasure prioritization based on causality, importance, and deviation-magnitude approaches following prediction of a temperature break by the LSGR. LPCMCI, using past 10 readings, recovered all (40) Temperature_Lagged (temperature lag) links during predicted break episodes, whereas CO_2_ links occurred predominantly (13) during normal operation. Across window lengths reported in Section 5.3.2.2, more links were recovered during break episodes than during normal operation for all features, indicating that the recovered structure differs by regime. Temperature_Lagged links dominated, consistent with the autocorrelation in Figure 1, indicating that the temperature trend itself is what needs to be controlled. Operationally, this structure is available at the point where a break is predicted rather than only after it occurs, providing a basis for informed intervention. Methodologically, causal discovery applied across an entire series would blend two distinct structures, so conditioning on regime is necessary. Characterizing this regime dependence more fully is left to future work.

##### 5.3.5.1 Balancing causality, importance and deviations

Depending on the range in which the predicted temperature falls, “Warning" (Table 8) on humidity and “critical" (Table 9) alerts, among others, were displayed, where *Cp*-val represents the causal *p*-value. These are based on Equation 29. Tables 9, 10 are PCMCI alerts presented for demonstration purposes. Table 8 shows that a feature (CO_2_) may be causal to the predicted break and within range, while its importance (SHAP) may be low, implying that causality is not correlation; neither does it imply a feature deviation, hence requiring no attention, while an out-of-range but non-causal feature (humidity) requires it. It also shows how a causal feature (temperature lag) is placed in a higher tier than a non-causal feature (humidity). Table 9 shows that a causal driver scores higher (above 15; the causal bonus is 15 in this study), while within a tier, the degree of deviation separates the deviated features: temperature lag score higher than humidity. This makes deviation magnitude important, as the most out-of-range feature needs urgent attention to avoid the predicted temperature break.

**Table 8:** Critical with causality and warning without it (Pred: 2.46, Actual: 2.3).

Feature	Input	SHAP	*Cp*-val	Causal
Temp. Lagged	2.200	–0.1207	0.048	✓
Humidity	0.850	0.0042	0.077	×
CO_2_	647.000	0.0029	0.030	✓
Ambient light	0.000	–0.0018	1.000	×
**Level**	**Feature**	**% Off**	**Change**	**Score**
Top alerts				
CRITICAL	Temp.	120.0	↓1.2 °C	15.9
WARNING	Humidity	6.0	↑0.05%	0.2

**Table 9:** Critical with causality (Pred: 2.35, Actual: 2.20).

Feature	Input	SHAP	*Cp*-val	Causal
Temp. Lagged	2.100	–0.1227	0.000	✓
Humidity	0.850	0.0034	0.007	✓
CO_2_	637.333	0.0023	0.804	×
Ambient Light	0.000	–0.0019	1.000	×
**Level**	**Feature**	**% Off**	**Change**	**Score**
Top alerts				
CRITICAL	Temp.	110	↓1.1 °C	16
CRITICAL	Humidity	6	↑ 0.05%	15.2

**Table 10:** Critical with and without causality (Pred: 27.28, Actual: 27.7).

Feature	Input	SHAP	*Cp*-val	Causal
Temp. Lagged	28.000	0.1440	0.708	×
Humidity	0.520	–0.0031	0.004	✓
Ambient Light	0.000	–0.0025	1.000	×
CO_2_	837.500	–0.0006	0.695	×
**Level**	**Feature**	**% Off**	**Change**	**Score**
Top alerts				
CRITICAL	Humidity	42	↑ 0.38%	15.5
CRITICAL	Temp.	2700	↓27 °C	0.8

Table 10 indicates that a higher SHAP (temperature lag) and degree of deviation do not translate to causality; hence, the need for prioritizing causality for control even if the feature (humidity) has a smaller deviation and importance, as causality provides useful causal information between the feature value and the predicted temperature break. By looking at Table 10, one may decide that temperature control should be prioritized since it should be reduced by a larger amount, which is not the case. Though causality-, importance-, and deviation-based threshold approaches differ, integrating these approaches with ML learning is a major contribution, given that a feature may be causal and important yet still within range; if controlled, it may fall outside the accepted range, resulting in temperature breaks. The harmonization of the approaches therefore provides a mechanism for valuing causality for a deviated feature upon an ML-predicted temperature break, but also balancing the importance and deviation if a causal feature is within range, informing relevant stakeholders about upcoming breaks and the conditions preceding the predicted break, how to counter them in advance, including the priorities of action. This enables transparent, trustworthy, proactive FFV temperature-abuse prevention, reducing wastage, enhancing food security, and preserving quality and safety across the cold chain.

##### 5.3.5.2 Feature prioritization method analysis

After a temperature break is predicted, causality (LPCMCI), SHAP, and threshold-based approaches (domain-based countermeasure prioritization using the magnitude of deviations) rank features for control prioritization. Though Section 5.3.5.1 presented alerts based on their weighted interactions, it is important to understand how they rank features. Figure 10 indicates that LPCMCI and SHAP, on average, agree on the ranking of temperature lag and ambient light. This is because past temperature is the most identified causal driver of predicted breaks and contributes much to the predictions. For ambient light, this is mainly because there are a few instances where it is important and causal, as shown in Figures 8, 9; hence, it is rarely ranked. Both methods' ranking agreement ranges between 46.73% and 54.13% at 95% confidence for temperature lag, humidity (22.68%, 29.16%), CO_2_ (28.04%, 34.91%) and ambient light (47.73%, 55.12%), indicating an average positional ranking agreement on features. This is supported by a Spearman correlation of 0.42. They have a top-2 agreement of 63.3%. This indicates general agreement in the top-2 parameter rankings, with temperature lag, followed by CO_2_ and humidity, appearing most frequently among the top two ranked parameters, thus increasing the chance of proactively controlling the key parameter first. However, these results point to the difference between correlation- and causality-based approaches on the importance or cause of predicted temperature breaks.

**Figure 10:**
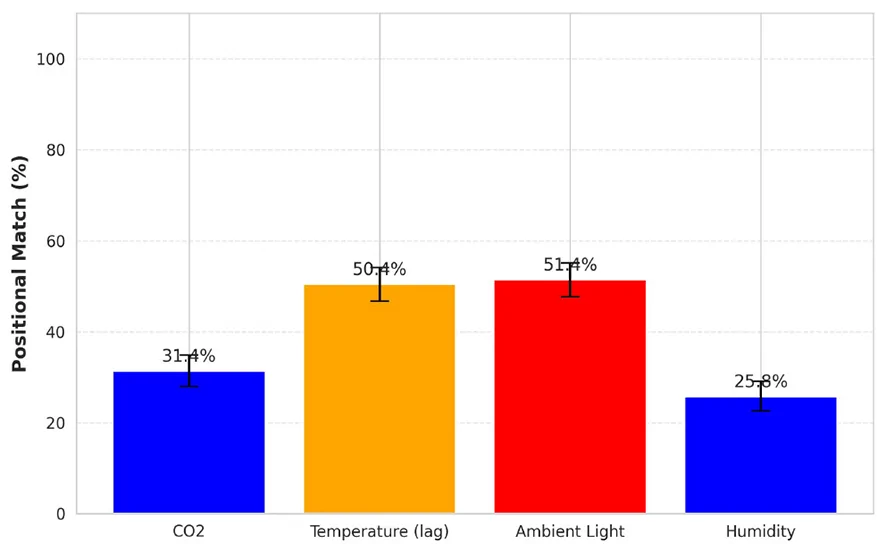
Feature ranking comparison post TBP: Causality vs SHAP.

From Figure 11, it can be seen that both causality and threshold-based approaches moderately agree on the positioning of temperature lag (63.96%, 70.91%) and humidity (56.81%, 64.80%), but not for ambient light (0.35%, 2.30%). This can be attributed to major deviations being for temperature lag, followed by humidity; hence, threshold attributes temperature to temperature breaks, while causality also found temperature lag crucial for explaining predictions. CO_2_ was excluded because it was uncommon since it did not deviate. Because humidity deviated a lot and had stronger causal strength than ambient light, both methods therefore agreed on its ranking. The correlation between their rankings is 0.226, implying they rarely agree on whether those variables are causes or correlates; hence, deviations do not always translate to causality. This, in principle, deviates from the common assumption that a feature that has deviated is simply the cause of a predicted deviation and vice versa, and the more the feature moves from the allowed range, the more causal it becomes for subsequent temperature values. On the contrary, both methods strongly agree on the ranking of the top-2 (irrespective of order) important parameters, temperature lag, and humidity (90.1%), which is key for proactively controlling the important parameter, as attending to either one first has a chance of avoiding the predicted break. Interestingly, SHAP importance and threshold approaches strongly agree on feature rankings with a correlation of 0.956 and a top-2 overlap of 90%. This is because temperature_lagged was highly important and deviated, as well as humidity, which ranked second in importance in the absence of CO_2_ ranking. This implies that a deviated feature comparatively contributes much to the predicted break.

**Figure 11:**
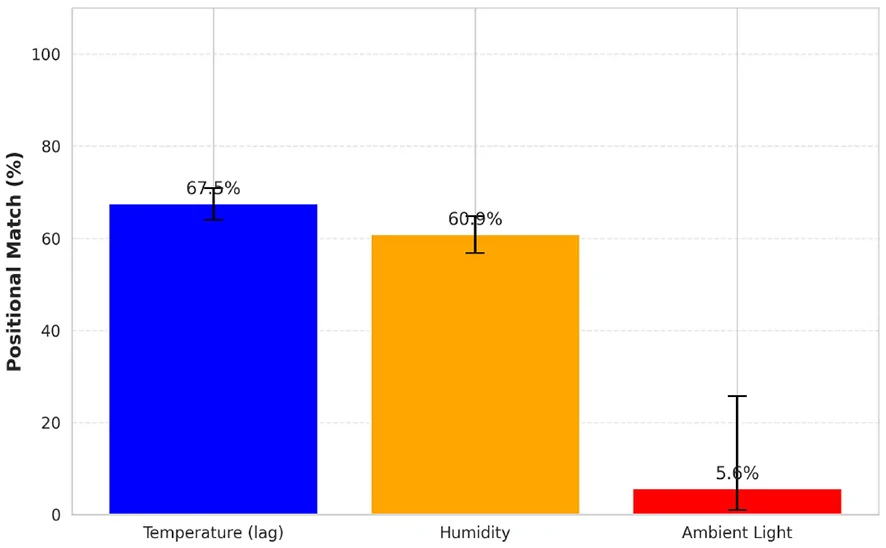
Feature ranking comparison post TBP: Causality vs Threshold.

Causality, importance and threshold approaches moderately agree on the ranking of temperature lag (63.66%, 70.63%) and humidity (56.10%, 64.11%), but strongly disagree for ambient light (0%, 17.59%). Therefore, they have an average rank correlation of 0.476 but a very high top-2 ranking of 89.5%. This is mainly because temperature lag ranked highly across the approaches, while the position of humidity was highly contested in terms of causality and importance. Based on the low rank correlation, it can be argued that the three disagree; hence, there is a need to find a balance, through weighting, between how features need to be prioritized based on all approaches, placing more importance on a more causal parameter; otherwise, deviations, if none of the measured features is causal. Since their top-2s are high, it may also be argued that the three approaches generally agree on the most critical features (Temperature_Lagged and Humidity), which is commendable, as all capture the key features which must be controlled to avoid or reduce the severity of temperature breaks, with a lower probability of fixing the least important parameter first. Again, this is a key contribution of this study, informing stakeholders, based on three different approaches, on the most critical factors responsible for temperature breaks in FFVs cold chains, from operators for focussed monitoring, and IT practitioners, for focussed modeling, including ML approaches, reducing costs associated for both stakeholders while providing a basis for improved cold chain integrity, reduced FFVs wastage, and associated impacts while increasing food security.

### 5.4 Limitations of the study

A primary limitation of this study is that causality based on LPCMCI and PCMCI depends much on domain knowledge to validate causal links. Additionally, by considering four measured parameters (temperature lag, relative humidity, ambient light, and CO_2_) for causality investigation, more causal links may have been missed. PCMCI and LPCMCI assume stable causal relationships across the analysis window. This was not tested. All experiments were run on a cluster. Memory use and whether the system could run on the low-resourced hardware typical of on-site monitoring were not tested. The sensitivity of the priority ranking to the chosen weights (Equation 27), causal bonus (Equation 26), and alert thresholds (Equation 29) was not assessed. The dataset used in this study covered July to December, spanning winter through early summer in a single cold room. Peak summer conditions and cross-room validation were not assessed, so transferability to other installations or to periods of higher ambient load remains untested.

## 6 Conclusion

This study addressed the challenge of temperature and break predictions in FFVs cold chain logistics, integrating ML with features' causality (LPCMCI, PCMCI) and importance (SHAP) to predicted temperature breaks, and based on apples' safety-based cold storage thresholds, to explain if a break will take place in the next hour, the conditions preceding it and how it can be avoided or have its severity reduced. The MLP, ELM, XGB, and tGRU from a conference paper, as well as the novel LSGR, sLSTM, bGRU, and bLSTM models, were evaluated on both smaller and larger IoT-gathered datasets. The ELM was the best across datasets, though slightly outperformed by persistence and AR(2) on EDS. However, the performance of the new DNN models improved on EDS, with LSGR being consistently accurate, suggesting that the deep models retained performance more consistently under the shift in operating conditions. Sensitivity analysis using LSGR identified temperature lag, humidity, CO_2_, ambient light and their interactions, a short historical window of a few past hours, and a three-to-one train-test split as critical for accurate temperature prediction, emphasizing careful feature selection and hyperparameter tuning in temporal deep learning models. On the extended dataset, the LSGR achieved the best overall performance for both temperature and temperature break predictions among the recurrent models assessed, with temperature predictions on average off by 0.9 °C and raising the fewest false alarms per day. The LSGR maintained shorter training times compared to most models, a key advantage for real-time cold chain monitoring. This affirms that combining LSTM's knowledge retention with GRU's training efficiency produces a robust and practical model for FFV cold chain applications, supporting better decision-making, reducing food wastage, and improving food security and profitability. All DNNs flagged breaks in advance, whereas persistence, recent mean, and AR(2) did not, indicating an operational capability the baselines lack. The proposed alert system leverages LSGR for TP, anticipated breaks, and their causal drivers, providing practitioners with prioritized countermeasures. LPCMCI outperformed PCMCI for causal discovery by effectively handling latent confounders. It discovered most causal links after temperature break prediction, especially for lagged temperature, identifying it as the dominant factor associated with predicted breaks. Despite disagreements in individual rankings, LPCMCI, SHAP, and threshold-based approaches consistently converged on temperature lag and humidity as the top two critical parameters, supporting the prioritization of countermeasure implementation. Future work should incorporate additional measured parameters and feature interactions into causal discovery, fuller characterization of causality-regime dependence, expand data collection across multiple features and seasons, and extend the framework to other cold chain phases including pre-cooling, transport, and retail. Model adaptability through incremental or online learning and edge- or fog-layer deployment are also recommended. Overall, this study equips cold chain practitioners with the tools to anticipate temperature breaks and their causal drivers, and proactively implement prioritized countermeasures, ultimately safeguarding FFV quality, reducing food wastage, and improving food security and profitability.

## Data Availability

The raw data supporting the conclusions of this article will be made available by the authors, without undue reservation. Additional data regarding the models optimization can be found in the Supplementary material.
