# Supplementary material for: Explainable deep learning for predicting temperature breaks and identifying their causal drivers in fresh produce cold chains
Source: Front Artif Intell. 2026 Oct 27;9:1893108. doi: 10.3389/frai.2026.1893108 (PMC13747965; doi:10.3389/frai.2026.1893108)
Supplement: Supplementary file 1 [file Supplementary_file_1.pdf]

Supplementary material for: Explainable deep learning for predicting temperature breaks and identifying their causal drivers in fresh produce cold chains

Jeremiah Taguta [1,*], **Jean Frederic Isingizwe Nturambirwe** [2,*] **and Clement Nthambazale Nyirenda** [1,2]

[1] *Department of Computer Science, University of the Western Cape, Cape Town, South Africa*
[2] *eResearch Office, University of the Western Cape, Cape Town, South Africa*

Correspondence*:
Corresponding Author
tagutaj@gmail.com, fisingizwe@uwc.ac.za

1 MACHINE LEARNING (ML) MODELS DESIGN

This section presents the search space and optimal parameters for the ML models used in this study.

1.1 ML models optimisation space

This section presents the search space for the ML models used in this study.

Table S1. Model Architectures, Hyperparameters, and Optimisation Strategies (Optim, Optimising algorithm; RS, Random search; GS, Grid search; ES, Early Stop)

Model	Key Hyperparameters	Optim	Splits
Conference Paper models			
XGB	`n_estimators`: [50–500], `max_depth`: [3–10], `learning_rate`: [0.01–0.3], `subsample ratio`: [0.5-1], `column sampling ratio`: [0.3-1]	GS	3-20
ELM	`hidden_neurons`: [5–200], `activation`: [lin, sigm, tanh, rbf_l2]	Manual	3-20
MLP	`layers`: [1–5], `units`: [32–512] + 32 step, `dropout`: [0–0.5], `optimiser`: [adam, rmsprop], `activation`: [relu, tanh, linear], BN	RS + ES	3-20
tGRU	`units`: [32–256], `activation`: [relu, tanh, sigmoid], `optimiser`: [adam, sgd, rmsprop], `batch_size`: [16, 32, 64], `epochs`: [50, 100, 200, 300, 400], `seq_length`: [10, 20, 60]	RS	3-20
This study's models (Novel) for Regression versus Classification			
tGRU	`units_0`: [50, 64, 128, 224, 256], `activation_0`: ['tanh', 'relu'], `dropout_0`: [0.0, 0.1, 0.2, 0.3, 0.4, 0.5], `use_batch_norm_0`: [True, False], `optimizer`: ['adam', 'rmsprop'], `learning_rate`: loguniform(1e-4, 1e-2), `patience`: [15, 20, 25], `batch_size`: [16, 32, 64]	RS + ES	

Table S1. (continued)

Model	Key Hyperparameters	Optim	Splits
DNNs	units_0: [50, 64, 128, 256], units_1: [50, 64, 128, 224, 256], activation_0: ['tanh', 'relu'], activation_1: ['tanh', 'relu'], dropout_0: [0.0, 0.1, 0.2, 0.3, 0.4, 0.5], dropout_1: [0.0, 0.1,0.2, 0.3, 0.4, 0.5], use_batch_norm_0: [True, False], use_batch_norm_1: [True, False], optimizer: ['adam', 'rmsprop'], learning_rate: loguniform(1e-4, 1e-2), patience: [15, 20, 25], batch_size: [16, 32, 64]	RS + ES	

1.2 ML models optimal parameters

This section presents the optimal parameters for the ML models used in this study.

Table S2. Model Architectures and Optimised Parameters (SL = Sequence length; BN = Batch Normalisation)

Model	Hyperparameters	SL	Splits
Conference Paper models			
XGB	n_estimators: 500, max_depth: 3, learning_rate: 0.05, subsample ratio: 0.5, column sampling ratio: 1		7
ELM	inputs: 14 , hidden_neurons: 14, output:1, activation: lin		3
MLP	480-unit dense, 0.3 dropout, 416-unit dense, 0.1 dropout layers and 1 dense layer layer, optimizer: rmsprop, activation: linear, epochs:100		7
tGRU	units: 256, BN, 1-unit dense, activation: tanh, optimiser: rmsprop, batch_size: 16, epochs: 300	10	7
This study's models (Novel) for Regression versus Classification			
tGRU	64-unit GRU, activation: tanh, recurrent_activation: sigmoid, BN, 1-unit dense, optimizer: rmsprop, learning_rate: 0.0002, batch_size: 64, patience: 20, epochs:200	2	3
LSGR	50-unit GRU, activation: tanh, recurrent_activation: sigmoid, 128-unit LSTM, activation: relu, recurrent_activation: sigmoid, BN, 0.2 dropout, 2-unit dense, 1-unit dense, optimizer: rmsprop, learning_rate: 0.004, batch_size: 32, patience: 25, epochs: 200	2	3
sLSTM	128-unit LSTM, activation: relu, recurrent_activation: sigmoid, 0.2 dropout, 256-unit LSTM, activation: tanh, recurrent_activation: sigmoid, 0.3 dropout, BN, 1-unit dense, optimizer: rmsprop, learning_rate: 0.004, batch_size: 32, patience: 25, epochs: 200	2	3
bGRU	50-unit Bidirectional GRU, activation: tanh, recurrent_activation: sigmoid, 0.5 dropout, 64-unit GRU, activation: relu, recurrent_activation: sigmoid, 0.2 dropout, BN, 1-unit dense, optimizer: rmsprop, learning_rate: 0.00075, patience: 25, batch_size: 16, epochs: 200	2	3
bLSTM	256-unit Bidirectional LSTM, activation: tanh, recurrent_activation: sigmoid, 0.3 dropout, 128-unit LSTM, activation: tanh, recurrent_activation: sigmoid, 0.5 dropout, BN, 1-unit dense, optimizer: adam, learning_rate: 0.005, patience: 25, batch_size: 32, epochs: 200	2	3

1.3 Feature groups

This section presents the feature groups used to analyse ML model's feature sensitivity.

Table S3. Feature Combinations for optimisation

ID	Features
A	[``Temperature'', ``Temperature_Lagged'', ``Temperature_CO2_Interaction'', ``Humidity_Temperature_Interaction'']
B	[``Temperature'', ``Humidity'', ``Average_Humidity'', ``Humidity_Lagged'', ``Humidity_Temperature_Interaction'', ``Humidity_CO2_Interaction'', ``Humidity_Variance'']
C	[``Temperature'',``CO2'', ``CO2_Lagged'', ``CO2_Trend'']
D	[``Temperature'', ``Ambient Light'', ``Ambient_Light_Trend'']
E	[``Temperature'', ``Acceleration_Magnitude'', ``Velocity_Magnitude'']
F	[``Temperature'',``Acceleration_Magnitude'', ``Velocity_Magnitude'', ``Acceleration (Y-axis)'', ``Velocity (Y-axis)'']
G	[``Temperature'', ``Temperature_Lagged'', ``Humidity_Temperature_Interaction'', ``Day_of_Month'', ``Temperature_CO2_Interaction'', ``Average_Humidity'',``Humidity_Lagged'', ``Humidity_CO2_Interaction'',``CO2_Trend'', ``Ambient Light'', ``Ambient_Light_Trend'', ``Humidity'', ``CO2'', ``CO2_Lagged'', ``Acceleration_Magnitude'', ``Velocity_Magnitude'']
H	[``Temperature'', ``Temperature_Lagged'', ``Humidity_Temperature_Interaction'', ``Temperature_CO2_Interaction'', ``Average_Humidity'', ``Humidity_Lagged'', ``Humidity_CO2_Interaction'', ``CO2_Trend'', ``Ambient Light'', ``Ambient_Light_Trend'', ``Humidity'', ``CO2'', ``CO2_Lagged'', ``Acceleration_Magnitude'', ``Velocity_Magnitude'']
I	[``Temperature'', ``Temperature_Lagged'', ``Humidity_Temperature_Interaction'', ``Day_of_Month'', ``Temperature_CO2_Interaction'', ``Average_Humidity'', ``Humidity_Lagged'', ``Humidity_CO2_Interaction'', ``CO2_Trend'', ``Ambient Light'', ``Ambient_Light_Trend'', ``Humidity'', ``CO2'', ``CO2_Lagged'']
J	[``Temperature'', ``Temperature_Lagged'', ``Humidity_Temperature_Interaction'', ``Day_of_Month'', ``Temperature_CO2_Interaction'', ``Average_Humidity'', ``Humidity_Lagged'', ``Humidity_CO2_Interaction'', ``CO2_Trend'', ``Ambient Light'', ``Ambient_Light_Trend'', ``Humidity'', ``CO2'', ``CO2_Lagged'', ``Acceleration_Magnitude'']
L	[``Temperature'', ``Temperature_Lagged'', ``Humidity_Temperature_Interaction'', ``Temperature_CO2_Interaction'', ``Average_Humidity'', ``Humidity_Lagged'', ``Humidity_CO2_Interaction'', ``CO2_Trend'', ``Ambient Light'', ``Ambient_Light_Trend'', ``Humidity'', ``CO2'', ``CO2_Lagged'', ``Acceleration_Magnitude'']
M	[``Temperature'',``Temperature_Lagged'', ``Humidity_Temperature_Interaction'', ``Acceleration_Magnitude'', ``Velocity_Magnitude'', ``Temperature_CO2_Interaction'', ``Average_Humidity'', ``Humidity'', ``Humidity_Lagged'', ``Velocity (Y-axis)'', ``Acceleration (Y-axis)'']

Continued on next page

Table S3 – continued

ID	Features
N	[``Temperature'', ``Temperature_Lagged'', ``Humidity_Temperature_Interaction'', ``Acceleration_Magnitude'', ``Velocity_Magnitude'', ``Temperature_CO2_Interaction'', ``Average_Humidity'', ``Humidity'', ``Humidity_Lagged'', ``Velocity (Y-axis)'', ``Acceleration (Y-axis)'', ``Day_of_Month'', ``Humidity_CO2_Interaction'', ``CO2_Trend'', ``Pressure'', ``CO2_Lagged'', ``CO2'']
O	[``Temperature'', ``Temperature_Lagged'', ``Humidity_Temperature_Interaction'', ``Acceleration_Magnitude'', ``Velocity_Magnitude'', ``Acceleration (X-axis)'', ``Velocity (X-axis)'', ``Acceleration (Z-axis)'', ``Temperature_CO2_Interaction'' , ``Velocity (Z-axis)'', ``Average_Humidity'', ``Humidity'', ``Humidity_Lagged'', ``Velocity (Y-axis)'', ``Acceleration (Y-axis)'']
P	[``Temperature'',``Temperature_Lagged'',``Humidity_Temperature_Interaction'', ``Acceleration_Magnitude'', ``Velocity_Magnitude'' , ``Acceleration (X-axis)'', ``Velocity (X-axis)'', ``Acceleration (Z-axis)'', ``Temperature_CO2_Interaction'', ``Velocity (Z-axis)'', ``Average_Humidity'', ``Humidity'', ``Humidity_Lagged'', ``Velocity (Y-axis)'', ``Acceleration (Y-axis)'', ``Day_of_Month'', ``Humidity_CO2_Interaction'', ``CO2_Trend'', ``Pressure'', ``CO2_Lagged'', ``CO2'']
Q	[``Temperature'', ``Temperature_Lagged'']
R	[``Temperature'', ``Temperature_Lagged'', ``Humidity_Temperature_Interaction'', ``Acceleration_Magnitude'', ``Velocity_Magnitude'', ``Acceleration (X-axis)'', ``Velocity (X-axis)'', ``Acceleration (Z-axis)'', ``Temperature_CO2_Interaction'' , ``Velocity (Z-axis)'', ``Average_Humidity'', ``Humidity'', ``Humidity_Lagged'', ``Velocity (Y-axis)'', ``Acceleration (Y-axis)'', ``Day_of_Month'', ``Humidity_CO2_Interaction'', ``CO2_Trend'', ``Pressure'', ``CO2_Lagged'', ``CO2'', ``Ambient_Light_Trend'', ``Ambient Light'', ``Temperature_Rate_of_Change'']
S	[``Temperature'', ``Temperature_Lagged'', ``Humidity_Temperature_Interaction'', ``Acceleration_Magnitude'', ``Velocity_Magnitude'', ``Acceleration (X-axis)'', ``Velocity (X-axis)'', ``Acceleration (Z-axis)'', ``Temperature_CO2_Interaction'', ``Velocity (Z-axis)'', ``Average_Humidity'', ``Humidity'', ``Humidity_Lagged'', ``Velocity (Y-axis)'', ``Acceleration (Y-axis)'', ``Day_of_Month'', ``Humidity_CO2_Interaction'', ``CO2_Trend'', ``Pressure'', ``CO2_Lagged'', ``CO2'', ``Ambient_Light_Trend'', ``Ambient Light'', ``Temperature_Rate_of_Change'',``Humidity_Variance'']
T	[``Temperature'', ``Temperature_Lagged'', ``Humidity_Temperature_Interaction'', ``Acceleration_Magnitude'', ``Velocity_Magnitude'', ``Acceleration (X-axis)'', ``Velocity (X-axis)'', ``Acceleration (Z-axis)'', ``Temperature_CO2_Interaction'' , ``Velocity (Z-axis)'', ``Average_Humidity'', ``Humidity'', ``Humidity_Lagged'', ``Velocity (Y-axis)'', ``Acceleration (Y-axis)'', ``Day_of_Month'', ``Humidity_CO2_Interaction'', ``CO2_Trend'', ``Pressure'', ``CO2_Lagged'', ``CO2'', ``Ambient_Light_Trend'', ``Ambient Light'', ``Temperature_Rate_of_Change'', ``Humidity_Variance'', ``Day_of_Week'', ``CO2_Rate_of_Change'']

Continued on next page

Table S3 – continued

ID	Features
U	[``Temperature'', ``Temperature_Lagged'', ``Humidity_Temperature_Interaction'', ``Temperature_CO2_Interaction'', ``Average_Humidity'', ``Humidity_Lagged'', ``Humidity_CO2_Interaction'', ``CO2_Trend'', ``Ambient Light'', ``Ambient_Light_Trend'', ``Humidity'', ``CO2'', ``CO2_Lagged'', ``Velocity_Magnitude'']
